# Minimal reduct for propositional circumscription

**DOI:** 10.3389/frai.2025.1614894

**Published:** 2025-12-10

**Authors:** Zhongtao Xie, Yisong Wang, Lei Yang, Renyan Feng

**Affiliations:** 1 State Key Laboratory of Public Big Data, Key Laboratory of Advanced Medical Imaging and Intelligent Computing of Guizhou Province, College of Computer Science and Technology, Guizhou University, Guiyang, China; 2 College of Mathematical Sciences, Minzu Normal University of Xingyi, Guiyang, China; 3 School of Information, GuiZhou University of Finance and Economics, Guiyang, China

**Keywords:** minimal reduct, propositional circumscription, satisfiability, answer set program, model-based diagnosis

## Abstract

Circumscription is an important logic framework for representing and reasoning common-sense knowledge. With efficient implementations for circumscription, including circ2dlp and aspino, it has been widely used in model-based diagnosis and other domains. We propose a notion of minimal reduct for propositional circumscription and prove a characterization theorem, *i.e*., that the models of a circumscription can be obtained from the minimal reduct of the circumscription. With the help of the minimal reduct, a new method circ-reduct for computing models of circumscription is presented. It iteratively computes smaller models under set inclusion (if possible), and the minimal reduct is used to simplify the circumscription in each iteration. The algorithm is proved to be correct. Extensive experiments are conducted on circuit diagnosis ISCAS85, random CNF instances, and some industrial SAT instances for the international SAT competition. These results demonstrate that the minimal reduct is effective in computing circumscription models. Compared to the widely used circumscription solver circ2dlp using the state-of-the-art answer set programming solver clingo, our algorithm circ-reduct achieves significantly shorter CPU time. Compared with aspino using glucose as the internal SAT solver and unsatisfiable core analysis technique, our algorithm achieves better CPU time for random and industrial CNF benchmarks, while it is comparable for circuit diagnosis benchmarks.

## 1 Introduction

Recent breakthroughs in large language models (LLMs) and foundation models have spurred an intense interest in integrating symbolic reasoning with data-driven learning to improve interpretability and controllable inference. Within this trend, classical logic techniques are being revisited as essential tools to provide strong guarantees and structured reasoning capabilities that purely statistical methods often lack (Ryu et al., 2025; Ye et al., 2023; Pan et al., 2023; Olausson et al., 2023; Callewaert et al., 2025).

Beyond classical approaches, non-monotonic reasoning has attracted renewed attention because it naturally captures default assumptions and common-sense knowledge that must be revised when new information arrives (Reiter, 1988). Recent ASP-based reasoning optimizations illustrate this synergy (Yang et al., 2023; Zeng et al., 2024; Rajasekharan et al., 2023). In addition, recent data-driven clustering methods continue to inspire logical inference. For example, automated cluster elimination guided by high-density points (Hu et al., 2025) and constrained clustering with weak label prior (Zhang et al., 2023) illustrate how structural knowledge can guide efficient search.

A prominent formalism for non-monotonic reasoning is circumscription, originally introduced by McCarthy for common-sense reasoning. Circumscription minimizes the extension of specific predicates, embodying a closed-world assumption where statements not known to be true are considered false (McCarthy, 1980). To increase the knowledge representation capabilities of ordinary circumscription, Lifschitz proposed parallel circumscription incorporating atoms that are allowed to vary (Lifschitz, 1985). Circumscription has received considerable attention in areas such as knowledge formalization (McCarthy, 1986; Lifschitz, 1986), common-sense reasoning (Wang et al., 2015; Alviano, 2019), diagnosis (Wotawa and Kaufmann, 2022; Metodi et al., 2014; Stern et al., 2012; Friedrich et al., 1999), planning (Sierra-Santibáñez, 2000), and privacy protection applications (Ogunniye and Kökciyan, 2023).

Despite its theoretical and practical significance, circumscription presents substantial computational challenges. It was shown that determining whether a circumscription has a model is NP-complete, verifying whether an interpretation is a model of a given circumscription is coNP-complete, and determining whether a formula is a logical consequence of a circumscription is $\Sigma_{2}^{p}$-complete (Eiter and Gottlob, 1993; Cadoli and Lenzerini, 1994). Consequently, efficiently computing models for propositional circumscription remains a formidable task. To the best of our knowledge, the main approach for computing circumscription models involves translating circumscriptions into (general) disjunctive logic programs under answer set semantics (ASP in short) (Gelfond and Lifschitz, 1988) such that the answer sets of the logic program correspond to the models of circumscription, allowing the use of efficient ASP solvers.

Sakama and Inoue (1995) proposed a method for translating circumscription into general disjunctive logic programs; however, this method requires the calculation of characteristic clauses, which can lead to exponential explosion. to translate finite first-order circumscription without varying predicates into first-order ASP. Although varying predicates can be eliminated in circumscription (Cadoli et al., 1992), the worst case can still result in exponential explosion. All the above translations for propositional circumscriptions either introduce fresh (predicate) symbols, or may result in exponential explosion. The ones for computing first-order circumscription primarily focus on translating first-order circumscription (or second-order theories) into first-order theories under certain constraints (Przymusinski, 1989; Doherty et al., 1997), for which there is no efficient implementation to the best of our knowledge.

Another alternative approach involves translating circumscription into the propositional satisfiability problem (SAT). Lee and Lin proposed a method that employs loop formulas and completions to translate circumscriptions into propositional theories. However, this method requires finding loop formulas, and the number of loop formulas may be exponential (Lee and Lin, 2006). Notably, enumerating models of circumscriptions is also interesting. For this purpose, Alviano proposed an unsatisfiable core analysis and implemented a solver aspino making use of the SAT solver glucose (Alviano, 2017), but this approach only employs solver which support cardinality constraints.

Recently, Wang et al. proposed the concept of minimal reduct for answer set programs. It substantially improves the minimal model decomposition of propositional theories (Angiulli et al., 2022), in addition to a new characterization for answer sets of logic programs (Zhang et al., 2021; Wang et al., 2023). Informally, the minimal reduct of a logic program *P* *w.r.t*. an interpretation *M* is obtained by replacing all atoms that are false under *M* in *P* with *false*.

In this study, we extend the notion of minimal reduct from answer set programming to (parallel) propositional circumscriptions and show that the minimal reduct preserves models of circumscription. With the help of minimal reduct and efficient SAT solvers, two approaches for computing and enumerating models of circumscriptions are presented, namely circ/circ-reduct and circ-enum. Extensive experimental results show that circ/circ-reduct is comparable with the state-of-the-art circumscription solvers circ2dlp and aspino.

The main contributions of this study are as follows:

- We introduce the concept of minimal reduct for propositional circumscription and establish its theoretical soundness by proving that it preserves satisfiability. Intuitively, the minimal reduct allows a circumscription formula to be simplified with respect to a given interpretation while keeping its essential models unchanged. This provides a new foundation for developing efficient SAT-based algorithms for computing circumscription models;
- We propose two sound algorithms for computing and enumerating models of propositional circumscriptions: circ, circ-reduct and circ-enum, respectively;
- We implement the aforementioned algorithms based on an open-source SAT solver (Cai et al., 2022) and conduct extensive experiments on diagnosis, random and industrial CNF formulas SAT competitions. We show that the two methods are comparable with the state-of-the-art methods circ2dlp and aspino.

The structure of the study is as follows: In Section 2, we review the necessary background knowledge; In Section 3, we propose minimal reduct for circumscription; In Section 4, we discuss the enumeration algorithm circ-enum; Section 5 details our experiments, demonstrating that the circ and circ-reduct methods can effectively compute propositional circumscription models; finally, we conclude the study and outline future research directions in Section 7.

## 2 Circumscription

In the section, we briefly recall the basic notions and notations of circumscription (McCarthy, 1980; Lifschitz, 1985; McCarthy, 1986):

Assume that the propositional symbols of the propositional language L form a finite set of atoms A. A literal *l* is either an atom *p* or the negation of an atom ¬*p*, while a clause α is a disjunction of literals:


$$\begin{array}{l} l_{1}\vee \cdots \vee l_{n}\;\left(n\geq 0\right) \end{array}$$ (1)

When *n* = 0, α≡⊥. For convenience, the clause α can also be written as the set of literals {*l*_1_, ⋯ , *l*_*n*_}. We denote $\alpha^{+}=A\cap \alpha$ and α^−^ = {*p*∣¬*p*∈α}. A clause theory *A* is a set of finite clauses.

If S⊆A, we denote $\bar{S}=A\backslash S$, ¬*S* = {¬*p*∣*p*∈*S*}, ∨*S* = ∨_*p*∈*S*_*p*, and ∧*S* = ∧_*p*∈*S*_*p*. The notions of formula, theory, interpretation, model, satisfaction (⊧), non-satisfaction (⊭), equivalence (≡), etc., in the propositional language L are the same as in classical propositional logic. We denote *uar*(*e*) as the set of all atomic symbols appearing in the formula (or theory) *e*.

Abusing the notation, a tuple (*t*_1_, …, *t*_*k*_) is usually written as {*t*_1_, …, *t*_*k*_} when there is no confusion from its context. The expression *A*(*P, Q*) denotes a formula/theory that contains atoms from *P*∪*Q*, where *P, Q* are disjoint sets of atoms; we denote *A*(*P*) as *A*(*P, Q*) when *Q* = ∅. When *X, Y* are tuples of the same length as *P, Q* respectively, *A*(*X, Y*) denotes the formula (or theory) obtained from *A* by simultaneously replacing the atoms from *P, Q* with the corresponding atoms from *X, Y*.

Example 1. Let *A*({*p, q*}, {*r*}) = (*p*∨¬*q*)∧(*r*∨¬*p*). Then *A*({*x, y*}, {*z*}) = (*x*∨¬*y*)∧(*z*∨¬*x*);

Let *P* = {*p*_1_, …, *p*_*n*_} and *Q* = {*q*_1_, …, *q*_*n*_} be two sets of atoms.

- *P* ≤ *Q* denotes ∧_1 ≤ *i* ≤ *n*_(*p*_*i*_→*q*_*i*_);
- *P* = *Q* denotes ∧_1 ≤ *i* ≤ *n*_(*p*_*i*_↔*q*_*i*_);
- *P*<*Q* denotes (*P* ≤ *Q*)∧¬(*P* = *Q*).

The basic idea of circumscription is to minimize the set of atoms assigned true as much as possible while keeping certain atoms fixed, thereby obtaining a “minimal” model.

Definition 1. Let *A* be a formula, and let *P, Z* be disjoint sets of atoms. The *circumscription* of *P* in formula *A* with *Z* allowed to vary, denoted as *CIRC*[*A*; *P*; *Z*], is the following formula:


$$\begin{array}{l} A\left(P,Z\right)\wedge \neg \exists XY\left(A\left(X,Y\right)\wedge \left(X<P\right)\right) \end{array}$$ (2)

where *X, Y* are fresh disjoint tuples of atoms with the same length as *P, Z* respectively. When *Z* = ∅, *CIRC*[*A*; *P*; *Z*] is shortened as *CIRC*[*A*; *P*].

In Definition 1, the *P* is called the *minimizing set*, the *Z* is called the *varying set*, and the set of remaining atoms is called the *fixing set*. Intuitively, the circumscription *CIRC*[*A*; *P*; *Z*] is to minimize (under set inclusion) the interpretation of *P*, while fixing the interpretation for $\bar{P\cup Z}$. The *varying set* *Z* consists of atoms whose truth values may change freely during this minimization, and as long as the original formula *A* remains satisfied, their assignment can be arbitrary.

Let *P* and *Z* be disjoint sets of atoms, *M, M*′ be two interpretations (sets of atoms). We use *M* ≤^*P*; *Z*^*M*′ to denote

- *M*∩*P*⊆*M*′∩*P*, and
- *M*\(*P*∪*Z*) = *M*′\(*P*∪*Z*).

If *M* ≤^*P*; *Z*^*M*′ holds but *M*′ ≤^*P*; *Z*^*M* does not, we denote this as *M*<^*P*; *Z*^*M*′. When *Z* = ∅, *M* ≤^*P*; *Z*^*M*′ is shortened to *M* ≤^*P*^*M*′, and *M*<^*P*; *Z*^*M*′ shortened to *M*<^*P*^*M*′.

Definition 2 (Circumscription model). Given a formula *A*(*P, Z*), for any interpretation *M*, if *M* is a model of *A*(*P, Z*) and there exists no model *M*′ of *A*(*P, Z*) satisfying *M*′ < ^*P*; *Z*^*M*, then *M* is a model of *CIRC*[*A*(*P, Z*);*P*; *Z*].

Intuitively, given a propositional formula φ, its circumscription in a set of minimizing atoms is the formula having only the models of φ that do not assign minimizing atoms to **true** unless necessary. For example, consider the formula *A* in Example 1, it is not hard to verify that {*p, q, r*}⊧*A* but {*p, q, r*}⊭*CIRC*[*A*; {*p, q, r*}] because ∅⊧*A* and ∅ < ^{*p, q, r*}^{*p, q, r*}. In fact, for any disjoint sets *P, Q* of atoms, ∅ is a model of *CIRC*[*A*; *P*; *Q*].

Example 2 (Circumscription in common-sense reasoning). Consider the use of circumscription in common-sense reasoning. Let


K={bird∧¬ab→fly},

which states that birds normally fly unless an abnormality (*ab*) occurs. When computing Circ[*K*; *ab*; *fly*], we obtain three models: {*bird, fly*}, {*fly*}, and ∅.

Here, *ab* is minimizing, meaning that we prefer situations where no abnormality occurs. Indeed, in all three models *ab* is false, which intuitively corresponds to “no abnormality happens.”

- The first model can be read as: it is a bird, and it flies.
- The second and third models correspond to situations where it is not a bird, in which case flying may or may not hold.

In this setting: *Fixing atoms* can be understood as conditions that partition the cases under consideration. *Minimizing atoms* typically represents abnormalities, which are assumed to be false unless evidence suggests otherwise. *Varying atoms* corresponds to reasoning outcomes that may change depending on the situation.

The next corollary easily follows from Definition 2.

Corollary 1. Let *A* be a formula and *M*⊆𝒜. Then

1. *M*⊧*CIRC*[*A*; *var*(*A*)] if and only if *M* is a minimal model of *A*.
2. *M*⊧*CIRC*[*A*; ∅; *var*(*A*)] if and only if *M*⊧*A*.
3. *CIRC*[*A*; *P*; *Z*] has a model if and only if *A* has a model.

The next example shows that circumscription is a generalization of the theory of minimal models.

Example 3. Let *A* = {*p*∨*q*}, 𝒜 = {*p, q*}. It's not hard to verify the following:

- *CIRC*[*A*; {*p, q*}] has two models {*p*} and {*q*}, that are exactly the minimal models of *A*.
- *CIRC*[*A*; {*p*};{*q*}] has only one model {*q*} since {*q*} < ^{*p*};{*q*}^ {*p*}.
- *CIRC*[*A*; ∅; {*p, q*}] has three models {*p*}, {*q*} and {*p, q*}.

## 3 Minimal reduct for circumscriptions

In this section, we introduce minimal reduct for circumscriptions. This reduct, based on the inherent structure of circumscription models, achieves efficient model search through iterative formula reduct and simplification.

Definition 3 (Minimal reduct). Given a clause theory *A*(*P, Z*) and *M*⊆𝒜, the *minimal reduct* of *A* w.r.t. *M*, *P* and *Z*,^1^ is the set of clauses consisting of, for each α∈*A*,


$$\begin{array}{l} \left(\alpha^{+}\cap \left(\left(P\cap M\right)\cup Z\right)\right)\cup \neg \left(\alpha^{-}\cap \left(\left(P\cap M\right)\cup Z\right)\right) \end{array}$$ (3)

such that

1. $\alpha^{-}\cap P\cap \bar{M}=\emptyset$,
2. $\alpha^{+}\cap \bar{P\cup Z}\cap M=\emptyset$, and
3. $\alpha^{-}\cap \bar{P\cup Z}\cap \bar{M}=\emptyset$.

The *minimal reduct* of the circumscription *CIRC*[*A*; *P*; *Z*] w.r.t. *M*⊆𝒜, denoted as *CRed*[*A*; *P*; *Z, M*], is the circumscription *CIRC*[*Red*(*A*; *P*; *Z, M*);*P*∩*M*; *Z*].

For convenience, we denote Equation 3 by *Red*[α; *P*; *Z, M*]. If one of the conditions (a), (b) or (c) in Definition (Equation 3) does not hold, then clause (Equation 3) does not belong to *Red*[*A*; *P*; *Z, M*]. In this case, we define *Red*[α; *P*; *Z, M*] = ⊤.

Thus, if there is no clause in *A* that satisfies one of the conditions (a), (b) and (c) then *Red*[*A*; *P*; *Z, M*]≡⊤.

Intuitively, the minimal reduct simplifies clauses with respect to a given interpretation. For a given clause and interpretation, if some atoms in the fixing set make the clause true, or if some atoms in the minimizing set that are **false** make the clause **true**, then the clause is reduced to ⊤. Otherwise, the clause is further simplified to retain only the varying atoms and those atoms in the minimizing set that are assigned **true**, so that minimization can continue on these relevant literals.

The next lemma follows from Definition 3 easily.

Lemma 1. Let *M*⊆𝒜, α(*P, Z*) be a clause, and *A*(*P, Z*) be a clause theory.

1. If *M*⊭α, then *Red*[α; *P*; *Z, M*] = α^+^∩*Z*∪¬(α^−^∩(*P*∪*Z*)).
2. *var*(*Red*[*A*; *P*; *Z, M*])⊆*P*∩*M*∪*Z*.

The following example illustrates the notions of minimal reduct *CRed* and *Red*.

Example 4. Let *P* = {*p, q*}, *Z* = {*z*}, *M* = {*q, a*}, *M*′ = {*p*}, *A*_1_(*P, Z*) consists of


$$\begin{array}{l} \alpha_{1}:\neg p\vee \neg z,\;\alpha_{2}:z\vee q,\;\alpha_{3}:\neg q\vee p\vee a. \end{array}$$

It is evident that *M*⊧*A*_1_ and $M^{\prime }⊭A_{1}$. We have:

- *Red*[*A*_1_; *P*; *Z, M*] = {*z*∨*q*}, because α_1_ does not satisfy the condition (*a*) of Definition 3, and α_3_ does not satisfy condition (*c*) of Definition 3.
- *CRed*[*A*_1_; *P*; *Z, M*] = *CIRC*[*z*∨*q*; {*q*};{*z*}], which has only one model {*z*}, and it is also a model of *CIRC*[*A*_1_; *P*; *Z*].
- $\text{Red}\left[A_{1};P;Z,M^{\prime }\right]=\left\{\neg p\vee \neg z,z\right\}$, which has a unique minimal model {*z*}; $\text{CRed}\left[A_{1};P;Z,M^{\prime }\right]=CIRC\left[\left\{\neg p\vee \neg z,z\right\};\left\{p\right\};\left\{z\right\}\right]$, which has only one model {*z*}.

**Intuitive explanation**. The distinction between fixing and minimizing atoms plays a crucial role here. For any *M*^⋆^ such that *M*^⋆^<^*P*; *Z*^*M*, the truth values of the *fixing* atom in {*a*} remain unchanged between *M*^⋆^ and *M*. For the *minimizing* atoms in {*p, q*}, if an atom is false in *M*, it must also be false in *M*^⋆^. The varying atom *z*, on the other hand, may freely change across models. These assumptions drive the simplification of clauses in the reduct.

Take *Red*[*A*_1_; *P*; *Z, M*] = {*z*∨*q*} as an example: For α_1_ = ¬*p*∨¬*z*, since *p* is false in *M*, ¬*p* is always true; hence α_1_ is satisfied and can be reduced to ⊤; For α_2_ = *z*∨*q*, the values of *z* and *q* cannot be determined from *M*, so the whole clause is retained; For α_3_ = ¬*q*∨*p*∨*a*, since *a* is true in *M*, the clause is always satisfied and reduces to ⊤.

Thus, only α_2_ survives in the reduct, yielding {*z*∨*q*}.

Next, we discuss the properties of minimal reduct. This lemma establishes the fundamental correspondence between a clause and its minimal reduct under a given interpretation M. It explains how the satisfaction of a reduced clause by an interpretation *M*′ reflects the satisfaction of the original clause by a combined interpretation that agrees with M on the fixing atoms while possibly minimizing atoms in P. In particular, Lemma 2 guarantees that the reduct construction preserves the semantic relationship between M and *M*′ with respect to the partial order ≤^*P*; *Z*^. This property is crucial for proving that the minimal reduct maintains the models of the original theory (Theorem 1) and, consequently, that the overall reduction process is sound with respect to the circumscription semantics.

Lemma 2. Let *P, Z, M* be the ones in Definition 3, the clause α satisfies conditions (*a*)-(*c*) in Definition 3, and *M*′⊆𝒜. Then

1. $M^{\prime }\cap \left(M\cap P\cup Z\right)\cup M\cap \bar{P\cup Z}\leq^{P;Z}M$;
2. α^−^∩(*M*∩*P*∪*Z*) = α^−^∩(*P*∪*Z*);
3. *M*′⊧*Red*[α; *P*; *Z, M*] if and only if $M^{\prime }\cap \left(M\cap P\cup Z\right)\cup \left(M\cap \bar{P\cup Z}\right)⊧\alpha$.

The next theorem shows that the minimal reduct for clause theories preserves the models of *A* that have the same assignment for fixing atoms, while the minimizing atoms may be minimized further.

Theorem 1. Let *A*(*P, Z*) and *M* be the ones in Definition 3, and *M*′⊆𝒜. Then *M*′⊧*Red*[*A*; *P*; *Z, M*] if and only if $M^{\prime }\cap \left(M\cap P\cup Z\right)\cup \left(M\cap \bar{P\cup Z}\right)⊧A$.

Informally, if a set of atoms *M*′ is a model of *Red*[*A*; *P*; *Z, M*], then one can construct a model of *A* that agrees with *M* on the fixing atoms and agrees with *M*′ on varying atoms, while the minimizing atoms in *M* are further minimizing in terms of *M*′.

Example 5. Let *M* = {*a, p, q*}, *A*(*P*; *Z*) = {¬*a*∨*p*∨¬*q, q*∨¬*p*}, where *P* = {*p, q*} and *Z* = ∅. It can be readily verified that *M* is a model of *A*(*P, Z*), and *Red*[*A*; *P*; *Z, M*] = {*p*∨¬*q, q*∨¬*p*}. The intersection $M\cap \bar{P\cup Z}$ is {*a*}. We consider the following two cases:

1. When *M*′ = ∅, we have *M*′⊧*Red*[*A*; *P*; *Z, M*]. Furthermore, $M^{\prime }\cap \left(M\cap P\cup Z\right)\cup \left(M\cap \bar{P\cup Z}\right)=\left\{a\right\}$, which is a model of *A*.
2. When *M*′ = {*p*}, we have *M*′⊭*Red*[*A*; *P*; *Z, M*]. The resulting set is $M^{\prime }\cap \left(M\cap P\cup Z\right)\cup \left(M\cap \bar{P\cup Z}\right)=\left\{a,p\right\}$, which is not a model of *A*.

The following theorem demonstrates how to construct a model of *CIRC*[*A*; *P*; *Z*] from a model *M*′of *CRed*[*A*; *P*; *Z, M*].

Theorem 2. Let *A*(*P, Z*) be a clause theory, and *M*′, *M*⊆𝒜. Then *M*′⊧*CRed*[*A*; *P*; *Z, M*] if and only if $M^{\prime }\cap \left(M\cap P\cup Z\right)\cup M\cap \bar{P\cup Z}⊧CIRC\left[A;P;Z\right]$.

Example 6. Let *A*(*P*; *Z*) = {*p*∨¬*q, q*∨¬*p, p*}, where *P* = {*p*} and *Z* = ∅. It is not hard to verify that *CIRC*[*A*; *P*; *Z*] has a unique model {*p, q*}. We consider the following cases:

1. Let *M*_1_ = {*p*}. Then *Red*[*A*; *P*; *Z, M*_1_] = {*p*, ¬*p*}, which is obviously unsatisfiable, so *CRed*[*A*; *P*; *Z, M*_1_] has no model. In fact, *CIRC*[*A*; *P*; *Z*] also has no such model *M* which assigns all fixing atoms **false**, *i.e*., $M\cap \bar{P\cup Z}=M\cap \left\{q\right\}=\emptyset$.
2. Let *M*_2_ = {*p, q*}. Then *Red*[*A*; *P*; *Z, M*_2_] = {*p*}, so *CRed*[*A*; *P*; *Z, M*_2_] has a unique model {*p*}. It's easy to see that {*p, q*}⊧*CRed*[*A*; *P*; *Z, M*_2_], and in fact, there is only one *M*′ such that $M^{\prime }\cap \left(M_{2}\cap P\cup Z\right)\cup M_{2}\cap \bar{P\cup Z}⊧CIRC\left[A;P;Z\right]$.

The next follows easily from Theorem 2.

Corollary 2. Let *A*(*P, Z*) be a clause theory, *Z* = ∅, *M, M*′⊆𝒜, *M* be a model of *A*(*P, Z*). The following statements are equivalent to each other:

i. *M*′⊧*CRed*[*A*; *P*; *Z, M*];
ii. *M*′ is a minimal model of *Red*[*A*; *P*; *Z, M*];
iii. $M^{\prime }\cap M\cap P\cup \left(M\cap \bar{P}\right)⊧CIRC\left[A;P;Z\right]$.

Note that a simple approach to compute circumscription models is to iteratively compute subset minimal models by calling an SAT solver in terms of Definition 1. With the help of Theorem 2, we obtain a new approach to compute circumscription models. It is based on the simple one and makes use of the minimal reduct to simplify the circumscription in each iteration. The typical one and the new one are named as circ and circ-reduct, respectively. They are presented as Algorithms 1, 2, while *Model*(.) extracts a model by calling an SAT solver which returns *unsat* if its input is unsatisfiable. This algorithm can be viewed as an extension of the works by Koshimura et al. (2009) and (Ben-Eliyahu and Dechter 1993). Specifically, when the fixing set is empty, this algorithm aligns with the methods described in Koshimura et al. (2009) and (Ben-Eliyahu and Dechter 1993).

Algorithm 1 circ(*A, P, Z*).

Require:  A circumscription *CIRC*[*A*; *P*; *Z*].
Ensure:  A model of *CIRC*[*A*; *P*; *Z*], and unsat otherwise.
1:  *M*←*Model*(*A*) ⊳ Computing a model of *A* by calling some SAT solver
2:  if *M* = unsat **then**
3:   return unsat
4:  end **if**
5:  $Y\leftarrow \left(M\backslash \left(P\cup Z\right)\right)\cup \neg \left(\bar{P\cup Z}\backslash M\right)$ ⊳ Fix assigments of fixing atoms
6:  while *M*∩*P*≠∅ **do**
7:   *T*←¬(*P*\*M*)∪{∨¬(*P*∩*M*)} ⊳ Constraint to be added for finding a smaller model under *P*
8:   *M*′←*Model*(*T*∪*A*∪*Y*)
9:   if *M*′ = unsat **then**
10:   break
11:   end **if**
12:   *M*←*M*′
13:  end **while**
14:  return *M*

Algorithm 2 circ-reduct (*A, P, Z*).

Require:  A circumscription *CIRC*[*A*; *P*; *Z*].
Ensure:  A model of *CIRC*[*A*; *P*; *Z*], and unsat otherwise.
1:  *M*←*Model*(*A*) ⊳ Computing a model of *A* by calling some SAT solver
2:  if *M* = unsat **then**
3:   return unsat
4:  end **if**
5:  *Y*←*M*\(*P*∪*Z*) ⊳ Retain the fixing atoms part of the model
6:  *A*′←*A*
7:  while *M*∩*P*≠∅ **do**
8:   *T*←{∨¬(*P*∩*M*)} ⊳ Constraint to be added for finding a smaller model on *P*
9:   *A*′←*Red*[*A*′; *P*; *Z, M*] ⊳ reduct the clause set by *P, Z, M*
10:   *M*′←*Model*(*T*∪*A*′)
11:   if *M*′ = unsat **then**
12:   break
13:   end **if**
14:   *M*←*M*′
15:  end **while**
16:  return *M*∪*Y* ⊳ Reconstruct the full model including fixing atoms

Intuitively, in Algorithm 1, the set *Y* determines the truth values of the fixing atoms of the model. Specifically, as long as *M*′⊧*Y*, the assignment of these atoms in *M*′ remains the same as in *M*. The set *T* ensures that fewer atoms in the minimizing set are assumed to be true. That is, *M*′∩*P*⊂*M*∩*P* if *M*′⊧*T*. Therefore, the main idea of this algorithm is to find an assignment with fewer true atoms in the minimizing set, while keeping the assignment of the fixing atoms, and satisfying the original theory *A*. Note that if the theory *A* is unsatisfiable, we assume that Model(A) returns unsat; otherwise, it returns a model of *A*. This algorithm calls the SAT solving procedure at most |*P*|+1 times in the worst case.

Theorem 3. Algorithm circ(*A*; *P*; *Z*) is correct. That is, if circ(*A, P, Z*) returns unsat, then there exists no model of *CIRC*[*A*; *P*; *Z*]; otherwise, the set of atoms returned by circ(*A, P, Z*) is a model of *CIRC*[*A*; *P*; *Z*].

The idea of Algorithm 1 is to compute circumscription models by adding block clauses, whereas Algorithm 2 computes them by reducing the size of the clause set. Clearly, Algorithm 2 also calls the SAT solver |*P*|+1 times in the worst case.

Theorem 4. Algorithm circ-reduct(*A, P, Z*) is correct. That is, circ-reduct(*A, P, Z*) returns a model of *CIRC*[*A*; *P*; *Z*] if *A* is satisfiable; otherwise, circ-reduct(*A, P, Z*) returns unsat.

The following example intuitively demonstrates the computation process of Algorithms 1, 2.

Example 7 (Continuation of Example 4). Here 𝒜 = {*p, q, z, a*}. Note that *P* = {*p, q*}, *Z* = {*z*}, and the clause theory *A*_1_(*P, Z*) consists of


$$\alpha_{1}:\neg p\vee \neg z,\;\alpha_{2}:z\vee q,\;\alpha_{3}:\neg q\vee p\vee a.$$

(a) The execution process of Algorithm 1 on *A*_1_, *P, Z* is as follows:

1. Assume that *Model*(*A*_1_) line 1 returns *M* = {*q, a*}, then at line 5, *Y* = {*a*};
2. In the first iteration of the while loop, *T* = {¬*p*}∪{¬*q*} (line 7);
  *M*′ = {*a, z*} (line 8, at this time the only model of *T*∪*A*∪*Y* is *M*′);
  *M*′≠ unsat (line 9);
  *M* = *M*′ (line 12);
  the loop ends (line 6), since *M*∩*P* = ∅;
3. At line 14, return *M* = {*z, a*}. It's easy to see that *M*⊧*CIRC*[*A*_1_; *P*; *Z*].

(b) The execution process of Algorithm 2 on *A*_1_, *P, Z* is as follows:

1. Assume that *Model*(*A*_1_) line 1 returns the model *M* = {*q, a*}, then at line 5, *Y* = {*a*};
2. In the first iteration of the while loop, *T* = {¬*q*} (line 8);
  *A*′ = {*z*∨*q*} (line 9);
  *M*′ = {*z*} (line 10, at this time the only model of *T*∪*A*′ is *M*′);
  *M*′≠ unsat (line 11);
  *M* = *M*′ (line 14);
  the loop ends (line 7), since *M*∩*P* = ∅;
3. At line 16, return *M* = {*z, a*}. It is easy to see that *M*⊧*CIRC*[*A*_1_; *P*; *Z*].

### 3.1 Complexity analysis

In this section, we analyze the time complexity of the two algorithms presented: Algorithm 1 (*circ*) and Algorithm 2 (*circ-reduct*). The complexity mainly depends on the number of SAT solver calls and the auxiliary set operations. Let the input clause theory *A* contain *n* clauses over *m* atoms. While propositional satisfiability has a worst-case complexity of *O*(2^*m*^), modern SAT solvers typically perform much better in practice due to sophisticated heuristics.

#### 3.1.1 Time complexity of algorithm (*circ*)

The complexity can be analyzed as follows:

- Initial SAT call: computing an initial model *M* of *A* requires one SAT solver call, in *O*(2^*m*^) time.
- Set operations: constructing *Y* by combining and negating subsets of *M* involves *O*(*m*) operations.
- Main loop: the loop iterates at most |*P*| times. In each iteration:
  - Constructing *T* takes *O*(|*P*|) time.
  - A SAT solver call on *T*∪*A*∪*Y* costs *O*(2^*m*^) time.

Thus, Algorithm 1 makes at most |*P*|+1 SAT calls, with polynomial overhead in *m*. The overall complexity is *O*(*m*·2^*m*^) since |*P*| ≤ *m*.

#### 3.1.2 Time complexity of algorithm *circ-reduct*

Algorithm 2 refines models by applying reducts until minimality is achieved:

- Initial SAT call: one SAT call on *A*, in *O*(2^*m*^) time.
- Set operations: constructing *Y* requires *O*(*m*) operations.
- Main loop: the loop executes at most |*P*| times. In each iteration:
  - Constructing *T* requires *O*(|*P*|) time.
  - Applying the reduct *Red*[*A*′; *P*; *Z, M*] requires at most *O*(*n*·*m*) operations.
  - A SAT solver call on *T*∪*A*′ runs in *O*(2^|*Z*|+|*M*∩*P*|^) time in the worst case.

Therefore, the loop contributes at most *O*(2^|*Z*|+|*P*|^) complexity. Including the initial SAT call, the total complexity is *O*(2^*m*^) since |*Z*|+|*P*| ≤ *m*.

Both algorithms are dominated by the number of SAT solver calls. Algorithm *circ* requires at most |*P*|+1 SAT solver calls, while Algorithm *circ-reduct* may involve additional clause reductions. In practice, modern SAT solvers significantly mitigate the theoretical exponential bound, and our empirical results confirm that the overhead of reduct operations is manageable.

## 4 Circumscription models enumeration

In this section, we further investigate the model enumeration problem for circumscriptions with the help of the algorithm circ and circ-reduct.

Let *P, Z* be disjoint finite subsets of 𝒜 and *M*⊆𝒜. By 𝒮(*M, P, Z*) we denote the formula


$$\begin{array}{l} \left(\vee \neg \left(M\backslash Z\right)\right)\vee \left(\vee \left(\bar{P\cup Z}\backslash M\right)\right). \end{array}$$ (4)

If *P* and *Z* are sets of “minimizing” and “varying” atoms, respectively, then the counter-models of constraint (Equation 4) correspond to the interpretations that are *P, Z*-greater than *M*, as shown by the next lemma. Thus, it can be used to exclude such *M* in the search for circumscription models.

Lemma 3. Let *P, Z* be disjoint finite subsets of 𝒜 and *M*⊆𝒜. Then, for each *M*′⊆𝒜, $M^{\prime }⊭S\left(M,P,Z\right)$ if and only if *M* ≤ *P*; *ZM*′.

Example 8. Consider the clause theory *A*(*P, Z*) = {α_1_:*p*∨*q*, α_2_:*p*∨*r*} where *P* = {*p, q, r*}, *Z* = ∅, and *M* = {*p*} We observe the following:

- S(M,P,Z)=¬p.
- For any *M*′ such that *p*∈*M*′, we have $M^{\prime }⊭S\left(M,P,Z\right)$ and *M* ≤ *P*; *ZM*′.

The next corollary easily follows from the above Lemma 3. It shows that the constraint (Equation 4) can indeed be used to compute some new circumscription models different on varying atoms.

Corollary 3. Let *A*(*P, Z*) be a clause theory, *M* be a model of *CIRC*[*A*; *P*; *Z*] and *M*′⊆𝒜. Then, $M^{\prime }⊧CIRC\left[A\cup \left\{S\left(M,P,Z\right)\right\};P;Z\right]$ if and only if *M*\*Z*≠*M*′\*Z* and *M*′⊧*CIRC*[*A*; *P*; *Z*].

According to Corollary 3, we propose the algorithm circWithZ to iteratively compute such models that are merely different on given varying atoms of *Z*. The following example shows how this algorithm is involved in computing such models.

Example 9. Let *A* = {α_1_:*p*∨*q*, α_2_:*a*∨¬*p*, α_3_:*q*∨*z*}, *P* = {*p, q*}, *Z* = {*z*}, and *M* = {*a, q, z*}. It is evident *M*⊧*A*. The computation process using circWithZ(*A, M, Z*) is as follows:

1. At line 1, *MS* = ∅;
2. At line 2, *Block* = {*a, q*, ¬*p*};
3. At line 3, *C* = {¬*z*};
4. At line 4 *A*′ = {*p*∨*q, a*∨¬*p, q*∨*z, a, q*, ¬*p*, ¬*z*};
5. In the first iteration of the while loop, *M*′ = {*a, q*} (line 6)
  *MS* = {{*a, q*}} (line 7);
  *C* = {*z*} (line 8);
  *A*′ = {*p*∨*q, a*∨¬*p, q*∨*z, a, q*, ¬*p*, ¬*z, z*} (line 9);
  the loop ends (line 5), since *A*′ is satisfiable;
6. At line 11 returns *MS* = {{*a, q*}}.

The correctness of the algorithm circWithZ is guaranteed by the next theorem.

Theorem 5. Algorithm 3 is sound and complete.

Algorithm 3 circWithZ(*A*,*M*,*Z*).

Require:  A clause theory *A* and *M, Z*⊆𝒜.
Ensure:  The set *MS* of models of *A* agreeing with *M* on $\bar{Z}$. 1:  *MS*←∅ 2:  $Block\leftarrow \left(M\cap \bar{Z}\cup \neg \left(\bar{Z}\cap \bar{M}\right)\right)$ ⊳ Fix the assignment of minimizing and fixing atoms

3:  *C*←(∨¬(*Z*∩*M*))∨(∨(*Z*\*M*)) ⊳ Clause excluding the current *Z*-assignment
4:  *A*′←*A*∪*Block*∪{*C*}
5:  while *A*′ is satisfiable **do**
6:   *M*′←*Model*(*A*′) ⊳ Computing a model of *A*′ by calling some SAT solver
7:   *MS*←*MS*∪{*M*′}
8:   *C*←(∨¬(*Z*∩*M*′))∨(∨(*Z*\*M*′)) ⊳ Exclude the new *Z*-assignment
9:   *A*′←*A*′∪{*C*}
10:  end **while**
11:  return *MS*

We are now in the position to present the algorithm of enumerating circumscription models, as shown by Algorithm 4. Informally, this algorithm circ-enum iteratively computes a circumscription model and then immediately enumerates all models that merely differ from the just-computed model. The core concept underlying this approach is akin to the strategies outlined in prior works, such as those by Dvořák et al. (2015) and (Niskanen and Järvisalo 2020).

Algorithm 4 circ-enum(*A*,*P*,*Z*).

Require:  A circumscription *CIRC*[*A*; *P*; *Z*].
Ensure:  The set *M* of all models of *CIRC*[*A*; *P*; *Z*].
1:  *MS*←∅
2:  while *A* is satisfiable **do**
3:   *M*←circ(*A, P, Z*) ⊳ Or equivalently call circ-reduct(*A, P, Z*)
4:   *M*_*z*_←circWithZ(*A, M, Z*) ⊳ Enumerate all variants under *Z*
5:   *MS*←*MS*∪{*M*}∪*M*_*z*_
6:   A←A∪{S(M,P,Z)} ⊳ Add blocking clause to exclude *M* and its *Z*-variants
7:  end **while**
8:  return *MS*

The correctness of circ-enum is guaranteed by the next theorem.

Theorem 6. Algorithm 4 is sound and complete.

The next example illustrates how circ-enum can be used to compute all circumscription models.

Example 10. Let *A* = {α_1_:*p*∨*q*, α_2_:*a*∨¬*p*, α_3_:*q*∨*z*}, *P* = {*p, q*}, and *Z* = {*z*}. The computation steps for circ-enum(*A, P, Z*) are detailed below:

1. In the first iteration of the while loop, we assume that circ (or circ-reduct) returns *M* = {*a, q, z*} at line 3, then
  *M*_*z*_ = {{*a, q*}} (line 4);
  *MS* = {{*a, q, z*}, {*a, q*}} (line 5);
  *A* = {*p*∨*q, a*∨¬*p, q*∨*z*, ¬*a*∨¬*q*} (line 6);
  enter next iteration of the while loop, since *A* is satisfiable;
2. In the second iteration of the while loop, we assume that circ (or circ-reduct) returns *M* = {*q, z*} at line 3, then
  *M*_*z*_ = {{*q*}} (line 4);
  *MS* = {{*a, q, z*}, {*a, q*}, {*q, z*}, {*q*}} (line 5);
  *A* = {*p*∨*q, a*∨¬*p, q*∨*z*, ¬*a*∨¬*q*, ¬*q*∨*a*} (line 6);
  the loop ends (line 2), since *A* is unsatisfiable;
3. At line 8 returns *MS* = {{*a, q, z*}, {*a, q*}, {*q, z*}, {*q*}}.

## 5 Experimental results

Based on one of the state-of-the-art SAT solvers LSTech-Maple,^2^ all three algorithms circ, circ-reduct, circ-enum have been implemented. This SAT solver integrates stochastic local search techniques into conflict-driven clause learning (CDCL) SAT solvers and keeps its completeness (Cai and Zhang, 2022; Cai et al., 2022).

To evaluate their performance, we selected three categories of benchmarks: model-based circuit diagnosis, random propositional circumscription, and various industrial test cases from the SAT competition.

We compare the three algorithms implemented circ and circ-reduct with aspino^3^ and circ2dlp^4^ The experimental environment includes a Linux server running Ubuntu 20.04 with an AMD EPYC 7742 CPU and 1007 GB of memory. The implementation and experimental data have been uploaded to GitHub.^5^

### 5.1 Note on unsatisfiable instances

For formulas that are unsatisfiable, the SAT solver invoked in the first step immediately reports UNSAT, so the core circumscription procedure is not executed. Consequently, the runtime and other performance metrics on such inputs are determined almost entirely by the underlying SAT solver, with the overhead of our circ/circ-reduct algorithms being negligible. UNSAT search itself can be costly, typically involving extensive branching and conflict analysis; approaches that extract unsatisfiable cores may introduce additional overhead, which likewise stems from the solver rather than our method.

In this section, we only foucs on CPU time, the memory will be reported in Supplementary material 2.

It should be emphasized that the issues observed with circ2dlp stem from minor implementation inconsistencies rather than from any flaw in the underlying theory of circumscription. In particular, circ2dlp may occasionally produce incorrect results when handling fixing atoms. For example, for *A* = {*x*_1_∨*x*_2_∨*x*_3_}, *P* = ∅, and *Z* = ∅, the rules generated from *A* by circ2dlp are not recognizable by clasp. Likewise, for *A* = {α_1_:*x*_1_∨*x*_3_, α_2_:¬*x*_1_∨*x*_2_} and *P* = {*x*_2_, *x*_3_}, circ2dlp returns only {*x*_3_} as the unique model of *CIRC*[*A*; *P*], whereas {*x*_1_, *x*_2_} is also a valid model. To handle such cases in practice, we developed the tool cnf2any,^6^ which eliminates fixing atoms following the method proposed by (de Kleer and Konolige 1989).

### 5.2 Integration with existing SAT frameworks

Our algorithms invoke an external SAT solver through its standard CNF interface. Modern SAT solvers such as Maple, Glucose, and MiniSat all provide C/C++ source code and well-documented APIs, so circ and circ-reduct can be integrated into other systems or embedded in larger software projects with minimal effort. This integration requires only routine engineering work–such as linking against the solver library or calling its incremental interface–and does not affect the theoretical correctness or performance guarantees of our approach. We emphasize that our implementation intentionally targets SAT solvers via CNF interfaces. Attempting to integrate the method with an ASP toolchain (e.g., clingo) is, in our assessment, not practically feasible without changing the problem or incurring prohibitive costs.

### 5.3 Model-based circuit diagnosis

Formally, a model-based circuit diagnosis is a triple *D* = (*SD, COMP, OBS*), where *SD* is a propositional theory that describes the circuit system, *COMP* is a finite set of components (atoms), and *OBS* is a set of literals representing system observations. A component set *M* is a *diagnosis* of *D* if and only if *SD*∪*OBS*∪{¬*Ab*(*a*)∣*a*∈*COMP*\*M*}∪{*Ab*(*c*)∣*c*∈*M*} is satisfiable, where *Ab*(*a*) indicates that component *a* is abnormal. If *M* is a diagnosis of *D* and no proper subset of *M* is a diagnosis of *D*, then *M* is referred to as a *minimal diagnosis* of *D* (Reiter, 1987).

We used 11 standard ISCAS85 circuits ^7^ as our benchmarks. These circuit descriptions are translated into CNF formulas that serve as the system description *SD*, the circuit gates as the components *COMP*, and random assignments of circuit inputs and outputs as the observations *OBS*. The minimal diagnoses of *D* = (*SD, COMP, OBS*) correspond to the circumscription models of *CIRC*[*SD*∪*OBS*; *COMP*]. It is important to note that all circuit components are the minimizing atoms of *CIRC*[*SD*∪*OBS*; *COMP*], while all other propositional symbols are treated as varying atoms of *CIRC*[*SD*∪*OBS*; *COMP*].

For each circuit, we randomly generated 20 observation instances, ran 5 times for each instance, with a CPU time limit of 1,800 s, and calculated the average CPU time for solved instances. The experimental results are presented in Table 1. The best CPU time for each circuit is in bold face. It can be seen that both circ and circ-reduct significantly outperform circ2dlp, and are comparable with aspino. In particular, while circ and circ-reduct solved all the 20 instances of the c6828 circuit, aspino ran out of 30 min for 19 instances. Additionally, the minimal reduct seems not very helpful for the benchmark since circ and circ-reduct have very similar average CPU time.

**Table 1:** The average CPU time in seconds for minimal circuit diagnosis.

**Instances**	**Circ**	**Circ-reduct**	**Aspino**	**Circ2dlp**
c17	**0.00018**	0.000211	0.000958	0.0123
c432	0.023595	0.022939	**0.002233**	–
c499	0.030602	0.032227	**0.008674**	–
c880	0.090575	0.087977	**0.004910**	–
c1355	0.160254	0.157518	**0.027911**	–
c1908	0.472890	0.502259	**0.280355**	–
c2670	0.532106	0.549403	**0.025147**	–
c3540	**1.530130**	1.613932	2.445828	–
c5315	1.746824	**1.672224**	2.904894	–
c6288	8.322000	**7.39054**	64.491870^*a*^	–
c7552	5.782249	5.669364	**2.869035**	–

^*a*^ There are 19 instances timeout.

–: Timeout.

Bold values denote the smallest CPU time among the compared solvers for each benchmark (i.e., the fastest solver).

### 5.4 Random circumscriptions

We examined our algorithms on random CNF instances whose number of atoms in 𝒜 ranges from 50 to 1000 with an interval of 50, and clause-to-atom ratios are from 3 to 5 with an interval of 0.5. The CNF instances were generated using the method proposed by Amendola et al. (2020). We generated 10 instances for each combination, resulting in 20 × 5 × 10 = 1000 random circumscriptions in total.

For each CNF φ over A, we created random circumscriptions *CIRC*[φ; *P*; *Z*] by randomly selecting disjoint subsets *P* and *Z* from A, with |*P*| and |*Z*| set to 10%, 30%, or 50% of |A|.

Tables 2, 3 present the average CPU time in seconds and the number of satisfiable and unsatisfiable instances solved by circ, circ-reduct, aspino, and circ2dlp within a 2-hour CPU time limit, respectively. It can be observed that both circ and circ-reduct significantly outperform aspino and circ2dlp by four orders of magnitude on satisfiable instances. Our methods solved a few hundred instances more than both aspino and circ2dlp.

**Table 2:** The number of solved satisfiable instances and the average CPU time in seconds for random circumscriptions.

|*P*|/|𝒜|	|*Z*|/|𝒜|	**# Solved satisfiable instances**				**Avg. CPU time (s)**			
		**Circ**	**Circ-reduct**	**Aspino**	**Circ2dlp**	**Circ**	**Circ-reduct**	**Aspino**	**Circ2dlp**
0.1	0.1	605	605	447	485	0.12	0.12	105.02	551.57
	0.3	605	605	455	485	0.13	0.13	161.86	349.15
	0.5	605	605	458	472	0.13	0.12	150.57	297.11
0.3	0.1	605	605	232	487	0.13	0.12	328.23	508.93
	0.3	605	605	233	424	0.12	0.13	278.90	3,445.2
	0.5	605	605	223	363	0.14	0.14	235.98	345.23
0.5	0.1	605	605	152	462	0.12	0.13	342.85	466.58
	0.3	605	605	152	364	0.12	0.13	267.34	381.35
	0.5	605	605	153	344	0.15	0.14	344.73	306.30

**Table 3:** The number of solved unsatisfiable instances and the average CPU time in seconds for random circumscriptions.

|*P*|/|𝒜|	|*Z*|/|𝒜|	**# Solved unsatisfiable instances**				**Avg. CPU time (s)**			
		**Circ**	**Circ-reduct**	**Aspino**	**Circ2dlp**	**Circ**	**Circ-reduct**	**Aspino**	**Circ2dlp**
0.1	0.1	172	170	160	139	366.60	308.47	433.19	376.22
	0.3	170	170	159	142	284.59	322.54	361.46	495.63
	0.5	171	170	156	140	336.75	307.47	315.77	321.30
0.3	0.1	170	170	157	142	300.84	311.12	324.04	497.34
	0.3	170	170	158	140	298.65	320.17	310.49	417.73
	0.5	172	170	157	142	362.98	310.19	339.42	431.81
0.5	0.1	171	171	157	141	336.08	361.29	316.01	466.71
	0.3	170	169	157	141	310.72	267.67	356.45	426.14
	0.5	171	170	158	141	340.94	296.64	372.04	398.69

For unsatisfiable instances, circ and circ-reduct also demonstrate an advantage by solving a greater number of instances, though there is no big difference in the average CPU time.

**Figure 1:**
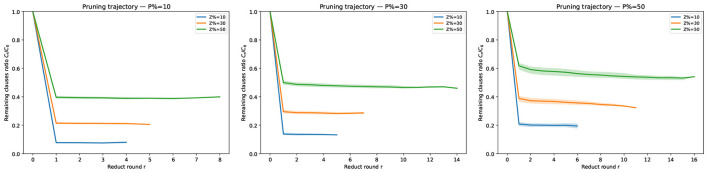
Per-round pruning trajectories by |*P*|/|𝒜|. Each panel fixes |*P*|/|𝒜| and shows the median remaining-clauses ratio *C*_*r*_/*C*_0_ across reduct rounds, with curves stratified by |*Z*|/|𝒜|∈{0.1, 0.3, 0.5}. Shaded bands indicate the interquartile range (IQR, 25th–75th percentile) across instances at each round; solid curves show the median. At round *r*, statistics aggregate only instances that reached at least *r* rounds.

**Figure 2:**
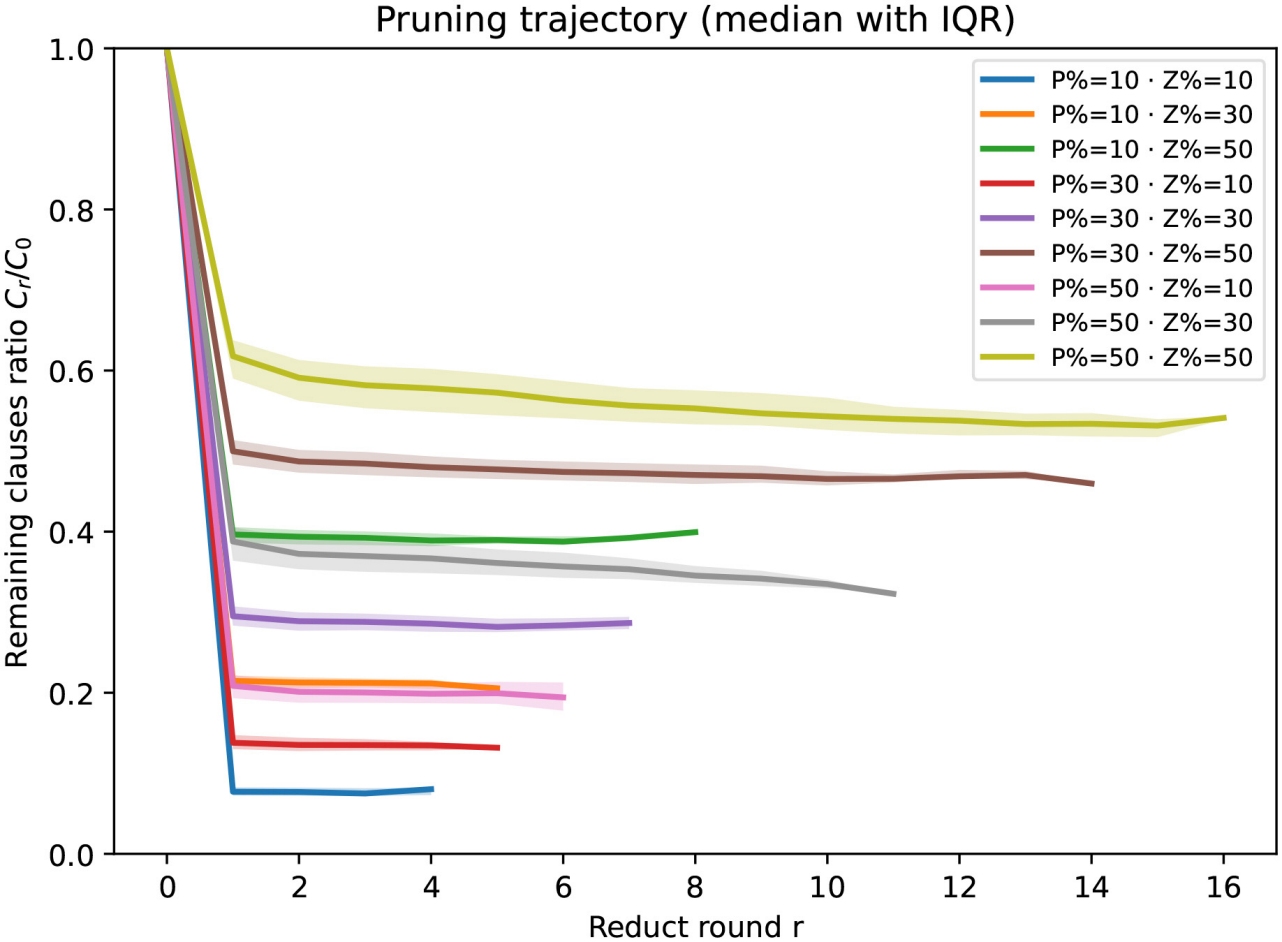
Overall pruning trajectories across (|*P*|/|𝒜|, |*Z*|/|𝒜|). Median remaining-clauses ratio *C*_*r*_/*C*_0_ with IQR bands (25th–75th percentile) across instances; lower curves indicate stronger pruning. Curves flatten more slowly as |*P*|/|𝒜| or |*Z*|/|𝒜| increases, indicating more reduct rounds before convergence.

Beyond average CPU time, we also analyze how the clause set shrinks across reduct rounds. For each circumscription instance we record the number of clauses *C*_*r*_ after round *r* and report the remaining-clauses ratio ρ_*r*_ = *C*_*r*_/*C*_0_ (lower is better). Since different instances may terminate after different numbers of rounds, the statistic at round *r* aggregates only those instances that reached at least *r* rounds (no imputation). Figure 1 shows per-round pruning trajectories split by |*P*|/|𝒜|∈{0.1, 0.3, 0.5} (three panels), with curves stratified by |*Z*|/|𝒜|∈{0.1, 0.3, 0.5}; bands indicate IQR across instances. Figure 2 overlays all nine (|*P*|/|𝒜|, |*Z*|/|𝒜|) settings in one panel for a global view.

Guided by the trajectories in Figures 1, 2, we observe:

1. Holding |*Z*|/|𝒜| (resp. |*P*|/|𝒜|) fixed, the total number of reduct rounds increases as |*P*|/|𝒜| (resp. |*Z*|/|𝒜|) grows, i.e., larger fixed or varying sets require more rounds before convergence. (ii) Across all configurations the **first** reduct yields the smallest remaining ratio (largest pruning step).
2. Quantitatively, after the first round we observe ρ_1_≈0.10 at (|*Z*|/|𝒜|, |*P*|/|𝒜|) = (0.1, 0.1) (about 90% pruned), whereas even in the least favorable setting (0.5, 0.5) the first round still leaves ρ_1_≈0.60 (about 40% pruned). These trends are consistent across all clause densities considered.

### 5.5 Circumscription of industrial CNF benchmarks

We choose moderate size industrial CNF benchmarks from SAT competitions.^8^ They are *Collatz* and *Johnson* from SAT-2019, *Giraldez* from SAT-2016, and *crypto* and *grieu* from SAT-2007. While both *Collatz* and *Johnson* contain 19 instances, *Giraldez* has 29 instances, *crypto* and *grieu* have 10 instances. To generate circumscription instances, we applied the same strategy to generate *P* and *Z* as described in Section 5.4. We report the metrics only for satisfiable instances, since there is no big difference for unsatisfiable instances, as shown in Table 3 for random CNFs.

Please note that while aspino, circ and circ-reduct specify varying and fixing predicates at the end of input files, circ2dlp specifies varying predicates and fixing predicates in the command line as arguments. Due to the very large number of atoms or clauses in *Collatz* and *crypto*, circ2dlp either outputs a “argument list too long” or runs out of time on the two benchmarks, while aspino always runs out of time. For clarity, the experimental results on *Collatz* and *crypto* are not reported in detail for aspino and circ2dlp. Tables 4–8 present the test results for the solved satisfiable instances by circ, circ-reduct, aspino, and circ2dlp in *Collatz, crypto, Johnson, Giraldez*, and *grieu* benchmarks, respectively. The experimental results further confirm the effectiveness of circ and circ-reduct compared with aspino and circ2dlp, while there is no much difference between circ and circ-reduct.

- There is no big difference between circ and circ-reduct in terms of the number of solved instances or the average CPU time per instance. Both circ and circ-reduct solved exactly the same number of satisfiable instances for all benchmarks excluding *Johnson*, for which circ-reduct solved slightly less number of instances than that of circ in at most 2. For the benchmark crypto the overall average CPU time of circ (resp. circ-reduct) is 840.37 (resp. 803.90), and for the benchmark Giraldez it is 63.77 (resp. 59.92). For the benchmark Callatz, the overall average CPU time of circ (resp. circ-reduct) is 1417 (resp. 1,437).
- circ and circ-reduct solved more instances than aspino and circ2dlp for the benchmarks *Johnson, Giraldez* and *grieu*. For the benchmark *Giraldez*, both circ and circ-reduct solve 18 instances, while aspino solved two instances, see Table 7. For the benchmark *Johnson*, circ and circ-reduct solved 5–7 instances, while circ2dlp solved up to two instances, see Table 6. There are similar results for the benchmark *grieu*, see Table 8.
- Both circ and circ-reduct have usually less average CPU time than aspino and circ2dlp for the solved instances of the benchmarks *Johnson, Giraldez* and *grieu*, even though circ and circ-reduct solved more instances.
- It seems that for all mentioned computing methods the ratios of minimizing and varying predicates over the signature do not have a distinguishing effect on the computing efficiency.

**Table 4:** The number of solved satisfiable instances and average CPU time in seconds for *Collatz*.

|*P*|/|𝒜|	|*Z*|/|𝒜|	**# Solved satisfiable instances**		**Avg. CPU time (s)**	
**Circ**	**Circ-reduct**	**Circ**	**Circ-reduct**
0.1	0.1	7	7	1,549.74	1,645.93
	0.3	8	8	1,376.15	1,396.52
	0.5	8	8	1,346.88	1,368.55
0.3	0.1	8	8	1,418.38	1,484.58
	0.3	8	8	1,342.50	1,421.92
	0.5	8	8	1,453.62	1,437.54
0.5	0.1	8	8	1,500.67	1,445.96
	0.3	8	8	1,362.92	1,305.51
	0.5	8	8	1,405.37	1,429.43

**Table 5:** The number of solved satisfiable instances, average CPU time in seconds for *crypto*.

|*P*|/|𝒜|	|*Z*|/|𝒜|	**# Solved satisfiable instances**		**Avg. CPU time (s)**	
		**Circ**	**Circ-reduct**	**Circ**	**Circ-reduct**
0.1	0.1	10	10	864.08	790.95
	0.3	10	10	848.10	837.96
	0.5	10	10	827.59	820.00
0.3	0.1	10	10	821.66	756.67
	0.3	10	10	862.14	824.34
	0.5	10	10	831.38	804.78
0.5	0.1	10	10	847.98	793.09
	0.3	10	10	822.27	806.67
	0.5	10	10	838.17	800.66

**Table 6:** The number of solved satisfiable instances and average CPU time in seconds for *Johnson*.

|*P*|/|𝒜|	|*Z*|/|𝒜|	**# Solved satisfiable instances**				**Avg. CPU time (s)**			
		**Circ**	**Circ-reduct**	**Aspino**	**Circ2dlp**	**Circ**	**Circ-reduct**	**Aspino**	**Circ2dlp**
0.1	0.1	6	6	2	0	1,874.11	1,836.31	630.27	–
	0.3	6	5	2	1	1,817.85	891.59	667.37	4,794.84
	0.5	7	6	2	2	2,462.56	1,984.04	661.55	5,804.20
0.3	0.1	7	5	4	1	2,497.59	900.14	3,264.90	4,181.70
	0.3	6	5	3	1	1,857.05	875.71	1,993.10	4,541.34
	0.5	7	5	4	1	2,523.02	889.17	3,325.13	4,911.33
0.5	0.1	7	5	3	1	2,431.79	866.36	3,107.61	3,986.31
	0.3	6	5	3	1	1,865.32	922.71	3,080.66	5,184.98
	0.5	7	6	3	1	2,588.24	1,916.91	2,783.58	4,909.26

–: timeout.

**Table 7:** The number of solved satisfiable instances and average CPU time in seconds for *Giraldez*.

|*P*|/|𝒜|	|*Z*|/|𝒜|	**# Solved satisfiable instances**				**Avg. CPU time (s)**			
		**Circ**	**Circ-reduct**	**Aspino**	**Circ2dlp**	**Circ**	**Circ-reduct**	**Aspino**	**Circ2dlp**
0.1	0.1	18	18	2	4	63.03	60.27	153.66	6,650.75
	0.3	18	18	2	11	58.11	60.07	118.12	5,203.65
	0.5	18	18	2	11	62.31	58.64	131.91	5,245.27
0.3	0.1	18	18	2	11	63.91	57.99	161.29	4,470.13
	0.3	18	18	2	11	66.90	59.33	160.48	5,100.03
	0.5	18	18	2	12	65.62	57.83	154.51	4,171.95
0.5	0.1	18	18	2	11	64.95	59.07	139.26	4,099.05
	0.3	18	18	2	11	63.88	57.39	109.29	4,645.46
	0.5	18	18	2	12	65.28	59.68	149.95	4,124.79

**Table 8:** The number of solved satisfiable instances and the average CPU time in seconds for *grieu*.

|*P*|/|𝒜|	|*Z*|/|𝒜|	**# Solved satisfiable instances**				**Avg. CPU time (s)**			
		**Circ**	**Circ-reduct**	**Aspino**	**Circ2dlp**	**Circ**	**Circ-reduct**	**Aspino**	**Circ2dlp**
0.1	0.1	10	10	6	5	753.31	853.19	894.55	1,185.86
	0.3	10	10	7	5	754.60	870.55	1,758.11	853.23
	0.5	10	10	6	5	712.04	878.16	936.14	851.03
0.3	0.1	10	10	5	5	765.83	824.98	581.69	850.02
	0.3	10	10	5	5	752.25	870.59	584.62	743.07
	0.5	10	10	5	5	720.84	873.77	625.78	739.57
0.5	0.1	10	10	5	5	731.53	860.21	1,713.07	634.19
	0.3	10	10	5	5	707.74	904.30	1,693.95	821.94
	0.5	10	10	4	5	735.66	959.91	2,147.24	607.86

For enumerating circumscription models, we have implemented the algorithm circ-enum calling circ or circ-reduct and evaluated it against all the above benchmarks. In this case, the CPU limit is set up to 30 min. The experimental results do not show a remarkable difference between circ-enum and aspino, while they outperform circ2dlp. For instance, circ2dlp enumerates all the 60 models for the diagnosis of circuit c17, aspino enumerates 0 models for the diagnosis of circuit c6288. For the random CNFs, aspino solved a larger number of instances than that of circ-enum. For the benchmark *grieu*, circ-enum computed more instances than aspino. We do not report these experimental results in detail for simplicity. Interested readers may refer to the experiments in the above github link.

## 6 Related work

Since the introduction of circumscription, the challenge of efficiently computing circumscription models has become a significant focus of research.

Currently, methods for computing circumscription are categorized into two classes: translating circumscriptions into logic programs (under stable model semantics) or leveraging SAT solvers.

### 6.1 Translating circumscription to logic programming

Early research concentrated on simplifying circumscription. Lifschitz (1985) proposed eliminating mutable predicates in circumscription in 1985, but this method introduced existential quantifiers. In 1988, Yuan and Wang extended this approach to specific cases, but both methods suffered from exponential growth (Yuan and Wang, 1988). Later, in 1992, Cadoli introduced a technique for eliminating variable predicates in circumscription (Cadoli et al., 1992); however, this method focused on inferring formulas from circumscription rather than transforming it. In 1989, Kleer and Konolige developed a method for eliminating fixing predicates in circumscription (de Kleer and Konolige, 1989).

In 1995, Sakama and Inoue proposed translating circumscription into a general disjunctive logic program (Sakama and Inoue, 1995), where the stable models correspond to circumscription models, but their method required the computation of characteristic clauses, leading to potential clause explosion. In 2008, Oikarinen and Janhunen addressed prioritized circumscription in the propositional case by translating it into a disjunctive logic program (Oikarinen and Janhunen, 2008), and implementing the tool circ2dlp. Similarly, in 2011, Gebser et al. developed metasp (Gebser et al., 2011), a general implementation technique using meta-programming, which reuses existing ASP systems to capture various forms of qualitative preferences among answer sets. This approach also enables the computation of circumscription in the propositional case without fixing predicates.

In 2014, Wan et al. developed cfo2lp, which translates first-order circumscription into a logic program, outperforming both circ2dlp and metasp on circuit diagnosis problem (Wan et al., 2014).

### 6.2 Translating circumscription to SAT

Inspired by loop formulas in computational logic programs (Lee and Lifschitz, 2003; Lin and Zhao, 2004), (Lee and Lin, 2006) proposed using loop formulas and completions to compute circumscription in the propositional case. However, the number of loops can grow exponentially, and the method requires eliminating existential quantifiers, making it potentially exponentially complex. In 2017, Alviano introduced an approach for enumerating propositional circumscription models using unsatisfiable core analysis, with the SAT solver functioning as a search engine (Alviano, 2017). This method also requires the introduction of auxiliary variables during computation.

In the propositional case, when the fixing predicate set is empty, the circumscription model is equivalent to the P-minimal model. Giunchiglia and Maratea (2006) proposed a tool in 2006 based on the Davis-Logemann-Loveland (DLL) algorithm to solve SAT-related optimization problems, including P-minimal models. In 2009, Koshimura introduced a SAT-based algorithm for computing P-minimal models (Koshimura et al., 2009). However, a common limitation of these approaches is their inability to handle circumscription containing fixing predicates.

A notable SAT-based approach relevant to our work is the counterexample-guided abstraction refinement (CEGAR) framework for propositional circumscription proposed by Janota et al. (2010). While their primary focus is on solving the entailment problem through iterative abstraction refinement, their CEGAR-based algorithm shares conceptual similarities with our constraint refinement process in Algorithms 1, 4. Specifically, both approaches employ counterexamples to guide the incremental refinement of constraints. In the context of circumscription, CEGAR excels at computing the set of variables assigned false in all models of a circumscribed formula.

### 6.3 Summary

Table 9 provides a comprehensive summary of the primary methodologies for computing circumscription models, delineating their core strategies and inherent limitations. Due to the absence of reported complexity analyses for certain methods, complexity metrics are excluded from the comparison. Furthermore, as the evaluation is restricted to comparisons with aspino and circ2dlp, performance metrics are not included. In general, the circ/circ-reduct approach demonstrates superior performance compared to aspino, while the circ and circ-reduct methods exhibit equivalent performance. Moreover, our proposed approach scales to significantly larger input instances than previously reported methods, highlighting its strong scalability.

**Table 9:** Comparison of methods for computing circumscription models.

**Method**	**Methodology**	**Strengths and limitations**
circ2dlp (Oikarinen and Janhunen, 2008)	ASP translation	Needs auxiliary vars.
aspino (Alviano, 2017)	SAT + unsat core analysis	Solver-dependent; only support for cardinality constraints
Loop formulas (Lee and Lin, 2006)	SAT + loop formulas	Exponential in number of loop formula
circ	SAT-based	No blowup; no aux./refresh vars.
circ-reduct	Minimal reduct + SAT-based	No blowup; no aux./refresh vars.
CEGAR (Janota et al., 2010)	Abstraction refinement	Computes vars. false in all models

## 7 Conclusion and future work

In this study, we introduced the notion of minimal reduct for circumscription and established a new characterization theorem for circumscription based on minimal reduct. We proposed and implemented two algorithms for computing models of circumscription: circ, which operates directly on propositional clause theories, and circ-reduct, which computes with the help of minimal reduct. We further presented the algorithm circ-enum to enumerate circumscription models.

We have evaluated our algorithms circ, circ-reduct and circ-enum on the benchmark model-based circuit diagnosis, random CNFs formulas, and several industrial benchmarks from SAT competitions. The results show that by employing the efficient SAT solver, both circ and circ-reduct are effective and significantly outperform circ2dlp and the solver based on unsatisfiable core analysis aspino. The former translates circumscriptions into disjunctive logic programs under stable model semantics. This study provides new directions for computing circumscription models.

The following issues deserve our further investigation:

- To optimize the model enumeration algorithm circ-enum for circumscription, we aim to draw inspiration from recent advancements in answer set programming (ASP) enumeration techniques (Calimeri et al., 2019; Comploi-Taupe et al., 2023). We plan to incorporate conflict-driven clause learning, backtracking, and heuristic search techniques. Additionally, given the connection between circumscription completion and loop formulas (Lee and Lin, 2006), we will explore how loop formulas can be fully utilized in enumerating circumscription models.
- Given a model of a circumscription, how to establish a justification for the atoms in the model. Similar ideas have been designed from answer set programming with the help of minimal reduct (Wang et al., 2023).
- Note that prioritized circumscription (Lifschitz, 1985) extends parallel circumscription by introducing priorities among minimizing predicates, which allows for the representation of priority-related applications. Given the existence of efficient computational methods for prioritized circumscription (Wakaki and Satoh, 1995, 1997; Chen, 1999; Oikarinen and Janhunen, 2008), we will consider how the notion of minimal reduct can be extended to prioritized circumscription. In particular, since prioritized circumscription can be reduced to the conjunction of multiple parallel circumscriptions, a natural preliminary direction is to apply the proposed minimal reduct approach sequentially to each component. Similarly, nested circumscription, which allows circumscription formulas themselves to appear as components, poses additional challenges. We leave the exploration of extending minimal reduct to nested circumscription as an interesting direction for future work.
- An interesting direction for future work is to extend our algorithms to parallel and distributed frameworks. Currently designed for sequential execution, the algorithms could benefit from parallelization, particularly during minimal reduct computation and model enumeration, which would significantly enhance scalability and efficiency for large-scale instances.

## Data Availability

The datasets presented in this study can be found in online repositories. The names of the repository/repositories and accession number(s) can be found in the article/Supplementary material.

## Footnotes

1 *P* and *Z* acts as the minimizing set of atoms and varying set of atoms, respectively.

2
https://github.com/shaowei-cai-group/LSTech-Maple

3
https://github.com/alviano/aspino

4
http://www.tcs.hut.fi/Software/circ2dlp/

5 https://github.com/gzu-ai/circ_solver, https://github.com/gzu-ai/circumscription_experiment

6
https://github.com/gzu-ai/cnf2any

7
https://sportlab.usc.edu/~msabrishami/benchmarks.html

8
http://satcompetition.org/
