# Supplementary material for: Minimal reduct for propositional circumscription
Source: Front Artif Intell. 2025 Dec 10;8:1614894. doi: 10.3389/frai.2025.1614894 (PMC12727910; doi:10.3389/frai.2025.1614894)
Supplement: Supplementary file 1 [file Supplementary_file_1.pdf]

Supplementary Material

1 EXPERIMENTS WITH VARIOUS SAT SOLVERS

To examine the solver-independence of our approach, we conducted additional experiments on two widely used SAT solvers, **Glucose** [1] and **Minisat** [2], in addition to the primary results obtained with LSTech-Maple2. All experiments were run on a Linux server with Ubuntu 20.04, equipped with an Intel® Xeon® Gold 5220 CPU and 511 GB of memory.

Table S1 reports the number of solved satisfiable instances and the average CPU time (in seconds) for the `circ` and `circ-reduct` algorithms on a set of minimal circuit diagnosis. The performance trends of both algorithms remain consistent across Glucose and Minisat, with only minor differences in runtime. These results confirm that the proposed methods are not tied to a specific SAT solver and can be effectively applied within different solver environments.

Table S1. Number of solved satisfiable instances and average CPU time (seconds) for minimal circuit diagnosis across different SAT solvers.

	Glucose		Minisat	
Instances	circ	circ-reduct	circ	circ-reduct
c17	0.000390	0.000403	0.000186	0.000207
c432	0.006133	0.005118	0.006777	0.006229
c499	0.006530	0.006421	0.007548	0.007754
c880	0.012292	0.012288	0.014206	0.014642
c1355	0.021023	0.022127	0.024661	0.028584
c1908	0.074393	0.074723	0.095893	0.103668
c2670	0.062867	0.063402	0.070537	0.073567
c3540	0.096716	0.087583	0.117234	0.108320
c5315	0.167347	0.164686	0.205608	0.192795
c6288	0.614795	1.121090	0.742104	1.378597
c7552	0.378038	0.404917	0.496461	0.491253

2 SUPPLEMENTARY MEMORY ANALYSIS

In addition to CPU time, we also measured the memory usage of the solvers on different benchmarks. Tables S2–S9 summarize the average memory consumption (in Mb) together with the number of solved instances.

Overall, the results show that `circ` and `circ-reduct` consistently consume more memory than `aspino` across almost all benchmarks. When comparing `circ` with `circ-reduct`, we observe that `circ` usually requires slightly more memory, although the difference is not uniform across all cases. These findings indicate that the proposed approaches, while competitive in CPU time, trade efficiency in memory usage for their broader applicability.

[1] https://github.com/audemard/glucose

[2] http://minisat.se/

Table S2. The average memory for minimal circuit diagnosis

Instances	circ	circ-reduct	aspino
c17	18.43242	15.88551	3.742773
c432	19.06690	19.06707	3.884962
c499	19.28923	19.30564	4.056054
c880	26.44331	25.14068	4.231835
c1355	33.29105	32.01567	4.687695
c1908	59.98178	57.52574	6.877147
c2670	74.54409	70.15426	5.454492
c3540	182.81995	164.05802	11.552733
c5315	201.23878	188.80316	10.351367
c6288	752.51593	634.62524	208.929102[1]
c7552	694.23533	554.13710	15.497268

[1] There are 19 instances timeout.
-: Timeout.

Table S3. The number of solved satisfiable instances and the average Memory for random circumscriptions

		# Solved Satisfiable Instances			Avg. Memory (Mb)		
$\|P\|/\|\mathcal{A}\|$	$\|Z\|/\|\mathcal{A}\|$	circ	circ-reduct	aspino	circ	circ-reduct	aspino
	0.1	605	605	454	19.22	16.08	10.87
0.1	0.3	605	605	448	19.58	16.37	9.96
	0.5	605	605	458	19.61	17.12	10.68
	0.1	605	605	217	19.92	16.65	16.51
0.3	0.3	605	605	234	19.95	17.47	15.86
	0.5	605	605	215	20.04	18.57	15.28
	0.1	605	605	165	20.04	17.24	12.52
0.5	0.3	605	605	161	20.08	18.62	13.81
	0.5	605	605	158	20.11	19.38	12.17

Table S4. The number of solved unsatisfiable instances and the average Memory for random circumscriptions

		# Solved Satisfiable Instances			Avg. Memory (Mb)		
$\|P\|/\|\mathcal{A}\|$	$\|Z\|/\|\mathcal{A}\|$	circ	circ-reduct	aspino	circ	circ-reduct	aspino
	0.1	172	170	149	41.41	39.60	14.41
0.1	0.3	170	170	148	39.62	39.62	14.48
	0.5	171	170	146	40.47	39.54	14.01
	0.1	170	170	147	39.66	39.68	13.43
0.3	0.3	170	170	147	39.59	39.58	13.72
	0.5	172	170	147	41.44	39.61	13.76
	0.1	171	171	143	40.56	40.97	13.06
0.5	0.3	170	169	147	39.59	38.80	14.18
	0.5	171	170	142	40.69	39.63	13.72

Table S5. The number of solved satisfiable instances and average memory for *Collatz*

		# Solved Satisfiable Instances		Avg. Memory (Mb)	
$\lvert P\rvert/\lvert\mathcal{A}\rvert$	$\lvert Z\rvert/\lvert\mathcal{A}\rvert$	circ	circ-reduct	circ	circ-reduct
	0.1	7	7	438.39	483.72
0.1	0.3	8	8	483.07	483.24
	0.5	8	8	478.47	476.97
	0.1	8	8	485.47	483.03
0.3	0.3	8	8	486.54	485.33
	0.5	8	8	481.52	479.93
	0.1	8	8	480.92	480.47
0.5	0.3	8	8	479.73	477.03
	0.5	8	8	515.66	497.98

Table S6. The number of solved satisfiable instances, average memory for *crypto*

		# Solved Satisfiable Instances		Avg. Memory (Mb)	
$\lvert P\rvert/\lvert\mathcal{A}\rvert$	$\lvert Z\rvert/\lvert\mathcal{A}\rvert$	circ	circ-reduct	circ	circ-reduct
	0.1	10	10	418.50	418.52
0.1	0.3	10	10	418.53	418.50
	0.5	10	10	419.26	419.19
	0.1	10	10	418.68	418.58
0.3	0.3	10	10	418.48	418.17
	0.5	10	10	418.90	418.95
	0.1	10	10	418.71	418.91
0.5	0.3	10	10	419.16	418.83
	0.5	10	10	420.38	419.39

Table S7. The number of solved satisfiable instances and average memory for *Johnson*

		# Solved Satisfiable Instances			Avg. Memory(Mb)		
$\lvert P\rvert/\lvert\mathcal{A}\rvert$	$\lvert Z\rvert/\lvert\mathcal{A}\rvert$	circ	circ-reduct	aspino	circ	circ-reduct	aspino
	0.1	6	6	2	171.84	205.44	88.49
0.1	0.3	6	5	2	175.08	171.28	88.49
	0.5	7	6	2	207.12	206.07	88.47
	0.1	7	5	2	205.56	171.28	36.37
0.3	0.3	6	5	2	176.78	173.33	33.77
	0.5	7	5	2	207.06	169.42	36.37
	0.1	7	5	1	205.63	171.33	36.39
0.5	0.3	6	5	1	176.80	169.42	36.35
	0.5	7	6	1	208.14	209.33	36.36

-: Timeout.

Table S8. The number of solved satisfiable instances and average memory for *Giraldez*

		# Solved Satisfiable Instances			Avg. Memory (Mb)		
$\vert P\vert/\vert\mathcal{A}\vert$	$\vert Z\vert/\vert\mathcal{A}\vert$	circ	circ-reduct	aspino	circ	circ-reduct	aspino
	0.1	18	18	2	41.84	42.06	33.47
0.1	0.3	18	18	2	42.11	42.10	34.16
	0.5	18	18	2	42.10	42.10	34.78
	0.1	18	18	2	42.11	42.10	33.94
0.3	0.3	18	18	2	41.86	41.86	35.56
	0.5	18	18	2	42.36	41.96	33.72
	0.1	18	18	2	41.88	42.11	33.48
0.5	0.3	18	18	2	42.37	42.12	35.09
	0.5	18	18	2	42.35	42.42	33.12

Table S9. The number of solved satisfiable instances and the average memory for *grieu*

		# Solved Satisfiable Instances			Avg. Memory (Mb)		
$\vert P\vert/\vert\mathcal{A}\vert$	$\vert Z\vert/\vert\mathcal{A}\vert$	circ	circ-reduct	aspino	circ	circ-reduct	aspino
	0.1	10	10	5	171.98	172.39	84.31
0.1	0.3	10	10	5	171.98	172.39	85.77
	0.5	10	10	5	170.93	171.34	88.05
	0.1	10	10	5	170.11	170.54	80.00
0.3	0.3	10	10	5	171.98	172.39	81.15
	0.5	10	10	5	170.11	170.54	80.01
	0.1	10	10	4	169.06	169.48	60.83
0.5	0.3	10	10	4	170.11	170.53	65.44
	0.5	10	10	4	171.98	172.40	60.95

3 FLOWCHARTS FOR ALGORITHMS

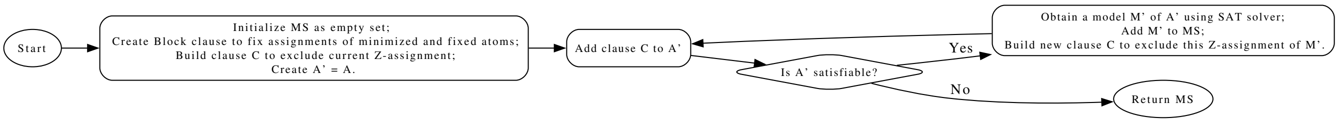

Figure S1. The flowchart of Algorithm 3 (circWithZ)

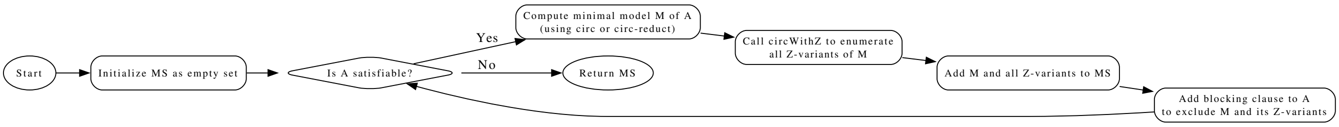

Figure S2. The flowchart of Algorithm 4 (circ-enum)

4 PROOFS

COROLLARY 1. *Let A be a formula and $M \subseteq \mathcal{A}$. Then*

1.$M \models CIRC[A; var(A)]$ *if and only if M is a minimal model of A.*

2.$M \models CIRC[A; \emptyset; var(A)]$ *if and only if* $M \models A$.

3.$CIRC[A; P; Z]$ *has a model if and only if A has a model.*

LEMMA 1. *Let $M \subseteq \mathcal{A}$, $\alpha(P, Z)$ be a clause, and $A(P, Z)$ be a clause theory.*

(i)If $M \not\models \alpha$, then $\textbf{\textit{Red}}[\alpha; P; Z, M] = \alpha^+ \cap Z \cup \neg(\alpha^- \cap (P \cup Z))$.

(ii)$var(\textbf{\textit{Red}}[A; P; Z, M]) \subseteq P \cap M \cup Z$.

PROOF. It is evident.

LEMMA 2. *Let P, Z, M be the ones in Definition 3, the clause α satisfies conditions (a)-(c) in Definition 3, and $M' \subseteq \mathcal{A}$. Then*

(i)$M' \cap (M \cap P \cup Z) \cup M \cap \overline{P \cup Z} \leq^{P;Z} M$*;*

(ii)$\alpha^- \cap (M \cap P \cup Z) = \alpha^- \cap (P \cup Z)$*;*

(iii)$M' \models \textbf{\textit{Red}}[\alpha; P; Z, M]$ *if and only if* $M' \cap (M \cap P \cup Z) \cup (M \cap \overline{P \cup Z}) \models \alpha$.

PROOF. Let $\beta = \textbf{\textit{Red}}[\alpha; P; Z, M]$.

(1) Obviously, $(M' \cap (M \cap P \cup Z) \cup M \cap \overline{P \cup Z}) \cap P = M' \cap M \cap P \subseteq M \cap P$ and $(M' \cap (M \cap P \cup Z) \cup M \cap \overline{P \cup Z}) \cap \overline{P \cup Z} = M \cap \overline{P \cup Z}$.

(2) ($\subseteq$) Obviously, $\alpha^- \cap (M \cap P \cup Z) \subseteq \alpha^- \cap (P \cup Z)$.

($\supseteq$) $\alpha^- \cap P \cap \overline{M} = \emptyset$ (condition (1) of Definition 3)
$\Rightarrow \alpha^- \cap P \subseteq M$
$\Rightarrow \alpha^- \cap P \subseteq M \cap P$
$\Rightarrow \alpha^- \cap (P \cup Z) \subseteq M \cap P \cup \alpha^- \cap Z$
$\Rightarrow \alpha^- \cap (P \cup Z) \subseteq \alpha^- \cap (M \cap P \cup Z)$.

(3) ($\Rightarrow$) $M' \models \beta$.
$\Rightarrow M' \cap (M \cap P \cup Z) \models \beta$ (by Lemma 1)
$\Rightarrow M' \cap (M \cap P \cup Z) \cup M \cap \overline{P \cup Z} \models \beta$ (by Lemma 1 and $\overline{P \cup Z} \cap var(\beta) = \emptyset$)
$\Rightarrow M' \cap (M \cap P \cup Z) \cup M \cap \overline{P \cup Z} \models \alpha$ (since $\beta \models \alpha$).

($\Leftarrow$) Assume $M' \not\models \beta$. Then we have
$M' \cap \beta^+ \cup (\beta^- \setminus M') = \emptyset$
$\Rightarrow M' \cap (M \cap P \cup Z) \cap \beta^+ = \emptyset$ and $\beta^- \subseteq M' \cap (M \cap P \cup Z)$ (Lemma 1)
$\Rightarrow M' \cap (M \cap P \cup Z) \cap \alpha^+ = \emptyset$ (since $\beta^+ = \alpha^+ \cap (M \cap P \cup Z)$) and
$\alpha^- \cap (M \cap P \cup Z) \subseteq M' \cap (M \cap P \cup Z)$ (since $\beta^- = \alpha^- \cap (M \cap P \cup Z)$)
$\Rightarrow (M' \cap (M \cap P \cup Z) \cup M \cap \overline{P \cup Z}) \cap \alpha^+ = \emptyset$ (condition (2) of Definition 3) and $\alpha^- \cap (P \cup Z) \subseteq M' \cap (M \cap P \cup Z)$ (by (2) of this lemma)
$\Rightarrow (M' \cap (M \cap P \cup Z) \cup M \cap \overline{P \cup Z}) \cap \alpha^+ = \emptyset$ and $\alpha^- \cap (P \cup Z) \cup \alpha^- \cap \overline{P \cup Z} \subseteq M' \cap (M \cap P \cup Z) \cup M \cap \overline{P \cup Z}$ (condition (3) of Definition 3)
$\Rightarrow (M' \cap (M \cap P \cup Z) \cup M \cap \overline{P \cup Z}) \cap \alpha^+ = \emptyset$ and $\alpha^- \subseteq M' \cap (M \cap P \cup Z) \cup M \cap \overline{P \cup Z}$
$\Rightarrow M' \cap (M \cap P \cup Z) \cup M \cap \overline{P \cup Z} \not\models \alpha$, which contradicts the condition.

THEOREM 1. *Let $A(P, Z)$ and M be the ones in Definition 3, and $M' \subseteq \mathcal{A}$. Then $M' \models$ **Red**$[A; P; Z, M]$ if and only if $M' \cap (M \cap P \cup Z) \cup (M \cap \overline{P \cup Z}) \models A$.*

PROOF. $M' \models$ **Red**$[A; P; Z, M]$
$\Leftrightarrow$ For any $\alpha \in A$ satisfying conditions (1)-(3) in Definition 3, $M' \models$ **Red**$[\alpha; P; Z, M]$
$\Leftrightarrow$ For any $\alpha \in A$ satisfying conditions (1)-(3) in Definition 3, $M' \cap (M \cap P \cup Z) \cup (M \cap \overline{P \cup Z}) \models \alpha$ (by (3) of Lemma 2)
$\Leftrightarrow$ For any $\alpha \in A$, $M' \cap (M \cap P \cup Z) \cup (M \cap \overline{P \cup Z}) \models \alpha$ (because for any $\beta \in A$ not satisfying any of the conditions (1)-(3) in Definition 3, $M' \cap (M \cap P \cup Z) \cup (M \cap \overline{P \cup Z}) \models \beta$)
$\Leftrightarrow M' \cap (M \cap P \cup Z) \cup (M \cap \overline{P \cup Z}) \models A$.

THEOREM 2. *Let $A(P, Z)$ be a clause theory, and $M', M \subseteq \mathcal{A}$. Then $M' \models$ **CRed**$[A; P; Z, M]$ if and only if $M' \cap (M \cap P \cup Z) \cup M \cap \overline{P \cup Z} \models CIRC[A; P; Z]$.*

PROOF. $M' \models$ **CRed**$[A; P; Z, M]$
$\Leftrightarrow M' \models CIRC[$**Red**$[A; P; Z, M]; P; Z]$
$\Leftrightarrow M' \models$ **Red**$[A; P; Z, M]$ and $\nexists M^* \models$ **Red**$[A; P; Z, M]$ s.t. $M^* <^{P;Z} M'$ (by Lemma 1)
$\Leftrightarrow M' \models$ **Red**$[A; P; Z, M]$ and $\nexists M^* \models$ **Red**$[A; P; Z, M]$ s.t. $M^* \cap P \subset M' \cap P$ and $M^* \cap \overline{P \cup Z} = M' \cap \overline{P \cup Z}$
$\Leftrightarrow M' \models$ **Red**$[A; P; Z, M]$ and $\nexists M^* \models$ **Red**$[A; P; Z, M]$ s.t. $M^* \cap P \subset M' \cap P$ (by Lemma 1, we can assume without loss of generality that $M^*, M' \subseteq M \cap P \cup Z$)
$\Leftrightarrow M' \cap (M \cap P \cup Z) \cup M \cap \overline{P \cup Z} \models A$ (by Lemma 2(3)), and $\nexists M^* \models$ **Red**$[A; P; Z, M]$ s.t. $M^* \cap P \subset M' \cap P$
$\Leftrightarrow M' \cap (M \cap P \cup Z) \cup M \cap \overline{P \cup Z} \models A$, and $\nexists M^*$ s.t. $M^* \cap (M \cap P \cup Z) \cup M \cap \overline{P \cup Z} \models A$ (by Lemma 2(3)) and $M^* \cap P \subset M' \cap P$

$\Leftrightarrow M' \cap (M \cap P \cup Z) \cup M \cap \overline{P \cup Z} \models A$, and $\nexists M^* \subseteq M \cap P \cup Z$ s.t. $M^* \cap (M \cap P \cup Z) \cup M \cap \overline{P \cup Z} \models A$ and $M^* \cap M \cap P \subset M' \cap M \cap P$ $(M' \subseteq M \cap P \cup Z)$
$\Leftrightarrow M' \cap (M \cap P \cup Z) \cup M \cap \overline{P \cup Z} \models A$, and $\nexists M^* \models\subseteq M \cap P \cup Z$ s.t. $M^* \cap (M \cap P \cup Z) \cup M \cap \overline{P \cup Z} \models A$ and $M^* \cap (M \cap P \cup Z) \cup M \cap \overline{P \cup Z} <^{P;Z} M' \cap (M \cap P \cup Z) \cup M \cap \overline{P \cup Z}$
$\Leftrightarrow M' \cap (M \cap P \cup Z) \cup M \cap \overline{P \cup Z} \models A$, and $\nexists M'' \models A$ s.t. $M'' <^{P;Z} M' \cap (M \cap P \cup Z) \cup M \cap \overline{P \cup Z}$
$\Leftrightarrow M' \cap (M \cap P \cup Z) \cup M \cap \overline{P \cup Z} \models CIRC[A; P; Z, M]$.

COROLLARY 2. *Let $A(P, Z)$ be a clause theory, $Z = \emptyset$, $M, M' \subseteq \mathcal{A}$, M be a model of $A(P, Z)$. The following statements are equivalent to one another:*

*(i)$M' \models$ **CRed**$[A; P; Z, M]$;*

*(ii)M' is a minimal model of **Red**$[A; P; Z, M]$;*

(iii)$M' \cap M \cap P \cup (M \cap \overline{P}) \models CIRC[A; P; Z]$.

PROOF. Let $P' = var($**Red**$(A; P; Z, M)) \cap P$.

(1) $\Leftrightarrow$ (2) $M' \models$ **CRed**$[A; P; Z, M]$
$\Leftrightarrow M' \models CIRC[$**Red**$(A; P; Z, M); P'; \emptyset]$
$\Leftrightarrow M'$ is a minimal model of **Red**$(A; P; Z, M)$ (by $Z = \emptyset$, Corollary 1(1) and Lemma 1).

(2) $\Leftrightarrow$ (3) M' is a minimal model of **Red**$[A; P; Z, M]$
$\Leftrightarrow M' \models CIRC[$**Red**$[A; P; Z, M]; P; \emptyset, M]$
$\Leftrightarrow M' \models$ **CRed**$[A; P; Z, M]$
$\Leftrightarrow M' \cap (M \cap P) \cup M \cap \overline{P} \models CIRC[A; P; Z, M]$ (Theorem 2).

THEOREM 3. *Algorithm $\texttt{circ}(A; P; Z)$ is correct. That is, if $\texttt{circ}(A, P, Z)$ returns $\texttt{unsat}$, then there exists no model of $CIRC[A; P; Z]$; otherwise, the set of atoms returned by $\texttt{circ}(A, P, Z)$ is a model of $CIRC[A; P; Z]$.*

PROOF. When circ(A, P, Z) returns unsat, it must be returned by the statement in line 4 of the algorithm, so A is unsatisfiable. By Corollary 1(3), $CIRC[A; P; Z]$ has no model.

When circ(A, P, Z) returns a set of atoms, it must be returned by the statement in line 16 of the algorithm.

First, in each non-terminating iteration of the loop, since $M' \models \neg(P \setminus M) \cup \{\bigvee \neg(P \cap M)\}$, each iteration makes at least one atom in $P \cap M$ assigned to be false in M' and keeps the atoms in P that are assigned to be false under the M still false in M'. Since $P \cap M$ is finite, this loop terminates after at most $|P|$ iterations.

In the first iteration of the WHILE loop when $M' \neq$ is unsatisfied, from $M' \models T$, we have:

- $M' \models A$ (because $M' \models T \cup A \cup Y)$),
- $M \cap \overline{P \cup Z} = M' \cap \overline{P \cup Z}$ (because $M' \models Y$), and
- $M' \cap P \subset M \cap P$ (because $M' \models \neg(P \setminus M) \cup \{\bigvee \neg(P \cap M)\}$).

Therefore, $M' <^{P;Z} M$.

When the WHILE loop terminates, either $M \cap P = \emptyset$ or T is unsatisfiable, meaning there does not exist $M'' \subseteq \mathcal{A}$ such that $M'' \models A$ and $M'' <^{P;Z} M'$.

By Definition 2, the set of atoms M returned by the algorithm is a model of $CIRC[A; P; Z]$. Therefore, this algorithm is correct.

THEOREM 4. *Algorithm* `circ-reduct`(A, P, Z) *is correct. That is,* `circ-reduct`(A, P, Z) *returns a model of* $CIRC[A; P; Z]$ *if* A *is satisfiable; otherwise,* `circ-reduct`(A, P, Z) *returns* `unsat`.

PROOF. (1) Clearly, circ-reduct(A, P, Z) returns unsat if and only if it returns from statement (line 3), in which case A is unsatisfiable. By Corollary 1 (3), when the algorithm returns unsat, $CIRC[A; P; Z]$ has no model.

(2) When algorithm circ-reduct(A, P, Z) returns a set of atoms, it must return from statement (line 16). In each non-terminating iteration of the WHILE loop, since $M' \models \neg(P \setminus M) \cup \{\bigvee \neg(P \cap M)\}$, i.e., M' keeps the atoms in $(P \setminus M)$ that are assigned to be false (because $M \models \neg(P \setminus M)$), and makes at least one atom in $P \cap M$ that is assigned to be false (because $M' \models \bigvee \neg(P \cap M)$), so $M' \cap P \subset M \cap P$; since $M \cap P$ is finite, this WHILE loop will definitely terminate.

Moreover, in the first iteration of WHILE when $M' \neq$ unsat, from $M' \models T \cup \mathit{Red}[A; P; Z, M]$, we have:

- $M' \cap (M \cap P \cup Z) \cup Y \models A'$ (by Theorem 1),
- $(M' \cap (M \cap P \cup Z) \cup Y) \cap \overline{P \cup Z}$
  $= (M' \cap (P \cup Z) \cup Y) \cap \overline{P \cup Z}$ (because $M' \models T$)
  $= Y \cap \overline{P \cup Z}$
  $= (M \setminus (P \cup Z)) \cap \overline{P \cup Z}$
  $= M \cap \overline{P \cup Z}$, and
- $(M' \cap (P \cup Z) \cup Y) \cap P = M' \cap P \subset M \cap P$ (because $M' \models T$).

Therefore, in each non-terminating iteration of the WHILE loop, $M' \cap (P \cup Z) \cup Y <^{P;Z} M$, and when the loop terminates, either $T \cup A'$ is unsatisfiable or $M \cap P = \emptyset$, so there does not exist M'' such that $M' \cap (M \cap P \cup Z) \cup Y \models A'$ and $M'' \cap (P \cup Z) \cup Y <^{P;Z} M'$. Thus, when the loop terminates, $M' \cap (P \cup Z) \cup Y \models CIRC[A; P; Z]$.

LEMMA 3. *Let* P, Z *be disjoint finite subsets of* $\mathcal{A}$ *and* $M \subseteq \mathcal{A}$. *Then, for each* $M' \subseteq \mathcal{A}$ *and* $M' \not\models \mathcal{S}(M, P, Z)$, *if and only if* $M \leq^{P;Z} M'$.

PROOF. Let $F = \mathcal{A} \setminus (P \cup Z)$, it's easy to verify that $\mathcal{A} = P \cup Z \cup F$.

$M' \not\models \mathcal{S}(M, P, Z)$
$\Leftrightarrow M' \not\models (\bigvee \neg (M \setminus Z)) \vee \bigvee \left(\overline{P \cup Z} \setminus M\right)$
$\Leftrightarrow M \setminus Z \subseteq M'$ and $\left(\overline{P \cup Z} \setminus M\right) \cap M' = \emptyset$
$\Leftrightarrow M \cap \overline{Z} \subseteq M'$ and $\left(\overline{P \cup Z} \setminus M\right) \cap M' = \emptyset$
$\Leftrightarrow M \cap (P \cup F) \subseteq M'$ and $(F \setminus M) \cap M' = \emptyset$ (since $\overline{Z} = P \cup F$)
$\Leftrightarrow M \cap P \subseteq M', M \cap F \subseteq M'$ and $(F \setminus M) \cap M' = \emptyset$ since $\overline{P \cup Z} = F$
$\Leftrightarrow M \cap P \subseteq M', M \cap F \subseteq M'$ and $F \cap \overline{M} \cap M' = \emptyset$
$\Leftrightarrow M \cap P \subseteq M', M \cap F \subseteq M'$ and $F \cap M' \cap \overline{M} = \emptyset$
$\Leftrightarrow M \cap P \subseteq M', M \cap F \subseteq M'$ and $F \cap M' \subseteq M$
$\Leftrightarrow M \cap P \subseteq M', M \cap F \subseteq M'$ and $F \cap M' \subseteq M \cap F$
$\Leftrightarrow M \cap P \subseteq M', M \cap F = F \cap M'$
$\Leftrightarrow M \cap P \subseteq M' \cap P$ and $M \cap \overline{P \cup Z} = \overline{P \cup Z} \cap M'$ (since $F = \overline{P \cup Z}$)
$\Leftrightarrow M \leq^{P;Z} M'$.

COROLLARY 3. *Let $A(P,Z)$ be a clause theory, M be a model of $CIRC[A;P;Z]$ and $M' \subseteq \mathcal{A}$. Then, $M' \models CIRC[A \cup \{\mathcal{S}(M,P,Z)\};P;Z]$ if and only if $M \setminus Z \neq M' \setminus Z$ and $M' \models CIRC[A;P;Z]$.*

PROOF. ($\Rightarrow$) $M' \models CIRC[A \cup \{\mathcal{S}(M,P,Z)\};P;Z]$
$\Rightarrow M' \models \mathcal{S}(M,P,Z)$
$\Rightarrow M \not\leq^{P;Z} M'$ (by Lemma 3)
$\Rightarrow M \setminus Z \neq M' \setminus Z$ (by Definition of $\leq^{P;Z}$)

Assume $M' \not\models CIRC[A;P;Z]$, then $\exists M_1 \models A$ s.t. $M_1 <^{P;Z} M'$ and $M \not\leq^{P;Z} M'$
$\Rightarrow M_1 \models A$ and $M_1 <^{P;Z} M'$ and $M \not\leq^{P;Z} M_1$
$\Rightarrow M_1 \models A \cup \{\mathcal{S}(M,P,Z)\}$ and $M_1 <^{P;Z} M'$ (by Lemma 3)
$\Rightarrow M' \not\models CIRC[A \cup \{\mathcal{S}(M,P,Z)\};P;Z]$

which contradicts $M' \models CIRC[A \cup \{\mathcal{S}(M,P,Z)\};P;Z]$.

($\Leftarrow$) $M \setminus Z \neq M' \setminus Z$ and $M' \models CIRC[A;P;Z]$
$\Rightarrow M \not\leq^{P;Z} M'$ and $M' \models A$ and $\nexists M_1 \models A$ s.t. $M_1 \not<^{P;Z} M$
$\Rightarrow M' \models \mathcal{S}(M,P,Z)$ and $M' \models A$ and $\nexists M_1 \models A$ s.t. $M_1 <^{P;Z} M$ (by Lemma 3)
$\Rightarrow M' \models A \cup \{\mathcal{S}(M,P,Z)\}$ and $\nexists M_1 \models A$ s.t. $M_1 <^{P;Z} M$
$\Rightarrow M' \models A \cup \{\mathcal{S}(M,P,Z)\}$ and $\nexists M_1 \models A \cup \{\mathcal{S}(M,P,Z)\}$ s.t. $M_1 <^{P;Z} M$
$\Rightarrow M' \models CIRC[A \cup \{\mathcal{S}(M,P,Z)\};P;Z]$

THEOREM 5. *Algorithm 3 is sound and complete.*

PROOF. Let M_i and M_j be the results of the i-th and j-th iterations of line 6 in Algorithm 3, respectively, where $i < j$. When $i(j) = 0$, $M_i(M_j) = M$.

We have $M_j \models (\bigvee \neg(Z \cap M_i)) \vee (\bigvee(Z \setminus M_i))$, and since $M_i \not\models (\bigvee \neg(Z \cap M_i)) \vee (\bigvee(Z \setminus M_i))$, therefore $M_i \neq M_j$.

Consequently, Algorithm 3 will terminate in a finite number of steps.

Let MS be the result returned by Algorithm 3 and $MS' = MS \cup \{M\}$.

For any $M' \in MS$, $M' \models \varphi$ (according to line 6) and $M' \setminus Z = M \setminus Z$ (according to line 2).

Assume there exists $M' \models A$ such that $M' \notin MS'$ and $M' \setminus Z = M \setminus Z$. Because $M' \notin MS'$, there exists $M_1 \in MS$ such that $M' \not\models (\bigvee \neg(Z \cap M_1)) \vee (\bigvee(Z \setminus M_1))$. Therefore, $M' = M_1$, which contradicts $M' \notin MS$.

In conclusion, Algorithm 3 is correct.

THEOREM 6. *Algorithm 4 is sound and complete.*

PROOF. Let MS be the result of Algorithm 4. Assume there exists $M \models CIRC[A;P;Z]$ such that $M \notin MS$.

Since $M \notin MS$, we have $M \not\models A \cup \bigcup_{M' \in MS} \mathcal{S}(M',A,P,Z)$ according to line 2.

Since $M \models CIRC[A;P;Z]$, $\exists M' \in MS$ such that $M \not\models \mathcal{S}(M',A,P,Z)$. According to Lemma 3, we have $M \leq^{P;Z} M'$. If $M <^{P;Z} M'$, this contradicts $M' \models CIRC[A;P;Z]$. Otherwise, M would be generated through line 4, which contradicts $M \notin MS$.
